# Evaluation of different deployment strategies for larviciding to control malaria: a simulation study

**DOI:** 10.1186/s12936-021-03854-4

**Published:** 2021-07-27

**Authors:** Manuela Runge, Salum Mapua, Ismail Nambunga, Thomas A. Smith, Nakul Chitnis, Fredros Okumu, Emilie Pothin

**Affiliations:** 1 Swiss Tropical and Public Health Institute, Basel, Switzerland; 2 University of Basel, Basel, Switzerland; 3 Environmental Health and Ecological Sciences Department, Ifakara Health Institute, Ifakara, Tanzania; 4 Clinton Health Access Initiative, Boston, USA

**Keywords:** Malaria, Larviciding, Larval source management, Mathematical modelling, OpenMalaria

## Abstract

**Background:**

Larviciding against malaria vectors in Africa has been limited to indoor residual spraying and insecticide-treated nets, but is increasingly being considered by some countries as a complementary strategy. However, despite progress towards improved larvicides and new tools for mapping or treating mosquito-breeding sites, little is known about the optimal deployment strategies for larviciding in different transmission and seasonality settings.

**Methods:**

A malaria transmission model, OpenMalaria, was used to simulate varying larviciding strategies and their impact on host-seeking mosquito densities, entomological inoculation rate (EIR) and malaria prevalence. Variations in coverage, duration, frequency, and timing of larviciding were simulated for three transmission intensities and four transmission seasonality profiles. Malaria transmission was assumed to follow rainfall with a lag of one month. Theoretical sub-Saharan African settings with *Anopheles gambiae* as the dominant vector were chosen to explore impact. Relative reduction compared to no larviciding was predicted for each indicator during the simulated larviciding period.

**Results:**

Larviciding immediately reduced the predicted host-seeking mosquito densities and EIRs to a maximum that approached or exceeded the simulated coverage. Reduction in prevalence was delayed by approximately one month. The relative reduction in prevalence was up to four times higher at low than high transmission. Reducing larviciding frequency (i.e., from every 5 to 10 days) resulted in substantial loss in effectiveness (54, 45 and 53% loss of impact for host-seeking mosquito densities, EIR and prevalence, respectively). In seasonal settings the most effective timing of larviciding was during or at the beginning of the rainy season and least impactful during the dry season, assuming larviciding deployment for four months.

**Conclusion:**

The results highlight the critical role of deployment strategies on the impact of larviciding. Overall, larviciding would be more effective in settings with low and seasonal transmission, and at the beginning and during the peak densities of the target species populations. For maximum impact, implementers should consider the practical ranges of coverage, duration, frequency, and timing of larviciding in their respective contexts. More operational data and improved calibration would enable models to become a practical tool to support malaria control programmes in developing larviciding strategies that account for the diversity of contexts.

**Supplementary Information:**

The online version contains supplementary material available at 10.1186/s12936-021-03854-4.

## Background

Larviciding is the application of biological or chemical insecticides that kill the immature stages of mosquitoes, and is one of the approaches of larval source management (LSM), along with habitat modification, habitat manipulation and biological control [1]. The World Health Organization (WHO) recommends larviciding as a supplementary intervention against malaria in addition to the core vector control interventions of insecticide-treated bed nets (ITNs) and indoor residual spraying (IRS) [2]. Larviciding is recommended in areas where the intervention is feasible and cost-effective, mostly in urban areas and during the dry season where breeding sites are ‘fixed, few and findable’ [1, 2]. Biolarvicides, *Bacillus thuringiensis israelensis* and *Bacillus sphaericus*, are currently the most prominent larvicides as they are environmentally safe [3]. However, under most environmental conditions, they have short residual effectiveness (*B. thuringiensis israelensis* lasts for only 1–2 weeks [7–9] and *B. sphaericus* for 2–3 weeks [6, 7]). Frequent applications have been widely recognized as a challenge for effective large-scale implementation [11–15].

Larviciding was widely used in the first half of the 20th Century, most successfully outside sub-Saharan Africa, but fell out of favour after the introduction of IRS with DDT [1, 13–16]. In the last decade, LSM, especially larviciding, has been reconsidered within an integrated vector management approach, especially as longer-lasting agents [17–19], or novel deployment strategies and breeding site identification, i.e., using drones [20, 21] might become increasingly available [22]. Post-2000, pilot programmes of larviciding have been conducted in urban and in rural areas in multiple countries of Africa [3, 9, 15, 21–39]. For example, the Urban Malaria Programme in Dar es Salaam, Tanzania, demonstrated operational feasibility and effectiveness of larviciding on larvae reduction and epidemiological outcomes in urban areas [11, 26]. In Burkina Faso, a trial in 84 rural villages with *B. sphaericus* applications during the main transmission season showed larviciding to be feasible and cost-effective when targeted to the most productive breeding sites [25, 43]. Pilot implementations have previously been included in national malaria strategic plans in Eritrea [7, 38, 44, 45], Zambia [31, 46, 47] and Nigeria [48]. Despite the long history of larviciding, its impact on malaria prevalence in humans [9, 49] in different settings, and the influence of variations in its application, particularly frequency and timing of the year as well as duration [6, 17, 40], remain insufficiently understood. For example, the application during the rainy season was described as impractical and less effective in study sites in Tanzania and The Gambia [26, 40], but as feasible in the study in Burkina Faso [24, 42], whereas its effectiveness during the dry season, as currently recommended, is still being debated [1, 16].

Mathematical models have been used to simulate mosquito population dynamics and the relationship between larval stages and adult mosquitoes [50–60]. However, most models consider only a small sub-set of the highly variable larviciding deployment scenarios. The models also include implicit assumptions about optimal deployment in relation to seasonality, (i.e., deployments either throughout the year, during rainy season or during dry seasons) and duration of larviciding effectiveness (i.e., constant or interrupted) without regard to re-treatment intervals and duration of product efficacy. While models have been used to simulate variations in the deployment strategies for other malaria control interventions, such as IRS [61–64] and drugs [64–67], larviciding strategies have not been investigated as much. In this study the impact of larviciding applications was simulated to assess the influence of different deployment strategies on expected entomological outcomes and malaria infections in humans for different seasonality and transmission settings.

## Methods

### Larviciding and influencing factors

**Fig. 1:**
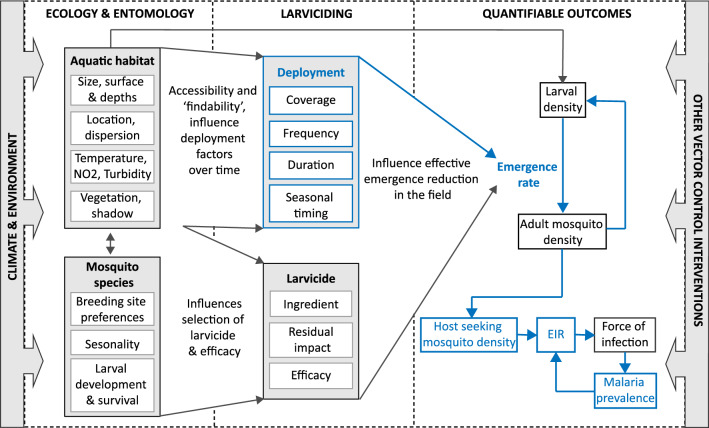
Flowchart of factors influencing larviciding effectiveness and malaria transmission outcomes. The flowchart reads from left to right, with climate and environmental factors influencing the whole system and relationships inside. This study focuses on the deployment factors and their impact on the quantifiable outcomes as highlighted in blue. All other factors were considered standard and non-varying

The application and effectiveness of larviciding is highly dependent on aquatic habitat characteristics and mosquito species [1]. Identification and accessibility of these habitats throughout the year is problematic yet essential [8, 11, 12]. Larviciding can reduce the number of emerging mosquitoes with a lag of two to three weeks between larviciding and reduction in adult mosquito density [4]. The number of infected host-seeking mosquitoes determines the entomological inoculation rate (EIR), which is related to the number of new infections in humans and the proportion of humans carrying malaria parasites. An overview of the most relevant influencing factors on larviciding application and effectiveness is shown in Fig. 1.

### Mosquito dynamics

A deterministic discrete-time model of overlapping generations of mosquitoes with a time step of one day was used [63, 64]. This was extended to include density-dependent larval dynamics using a Beverton-Holt formulation [71, 72]. This model includes a single juvenile stage of mosquitoes arising at a rate proportional to the time-lagged number of eggs laid, with the larval population regulated by periodically varying larval-carrying capacity and the larvae progressing to host-seeking adults at a constant rate. The assumed lag times between rainfall was 10 days for mosquito emergence, 20 days for host-seeking mosquitoes and 30 days to actual transmission events (measured as EIR). The models and derived parameters are described in Additional file 1. The entomological model was connected to a stochastic individual-based model for malaria in humans [74] within the OpenMalaria platform [68, 69, 74–76], for which the source code is available online [77]. The parasite densities per simulated infection vary by 5-day time steps.

### Model parameterization

Malaria transmission intensity was defined as annual pre-larviciding EIR and simulated with three intensity levels: 3 infectious bites per person per annum (ibpa) for low transmission, 10 ibpa for moderate transmission and 90 ibpa for high transmission. Seasonality was characterized as high or medium seasonal with either one or two peaks and reproduced from another modelling study [78]. The seasonality in transmission was assumed to follow the same pattern as the rainfall with a lag of one month, and lag time between key indicators are shown in Additional file 2: Fig. S2.1. The characteristics of the setting, including resistance, host preferences and biting behaviour were held constant over time. Differences between species were not considered, and a previously determined parameterization for *Anopheles gambiae *sensu stricto* (s.s.),* predominantly indoor biting and anthrophilic, was used. To explore sensitivity to highly uncertain mosquito population density-dependency parameters, the survival probability of larvae, the development duration and the number of female eggs per gonotrophic cycle were varied.

**Fig. 2:**
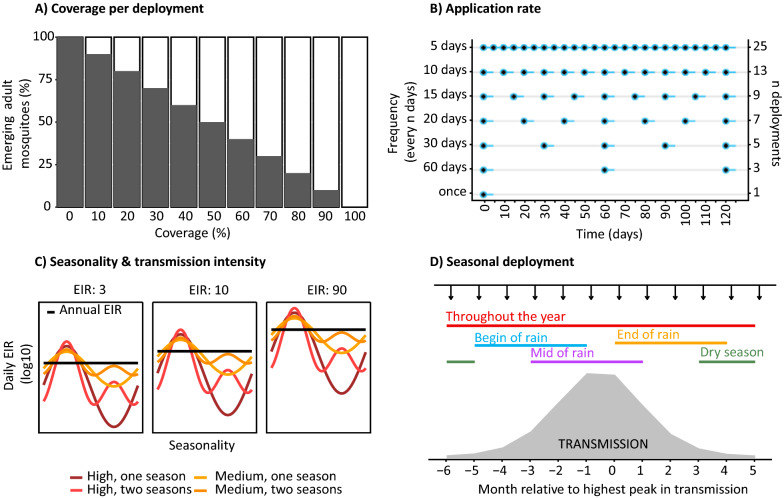
Illustration of setting and deployment specific parameter. **A** Larviciding coverage, held constant for all deployments. **B** Deployment frequency during one deployment period of 120 days. The deployment frequency is characterized by the number of deployments per unit of time (rectangles on the right) and characterizes the lengths of gaps in effective coverage between single deployments. The blue lines correspond to the duration of time the larvicide is active, assumed to be 5 days. The emergence rate is unaffected outside of these blue lines. **C** Transmission seasonality patterns reproduced from [78]. **D** Seasonal deployment times with larviciding starting at different months (12 independent scenarios) assuming a constant efficacy of 120 days, or 365 days in case of deployment ‘throughout the year’, included for comparison

Simulated parameters of larviciding included the coverage, deployment duration, application frequency, and seasonal deployment. The coverage was defined as the reduction in emerging mosquitoes as a result of treated breeding sites (operational coverage) and larvicide efficacy (Additional file 3). It was assumed that important aquatic habitats had been pre-identified and characterized, and that they were accessible and evenly distributed. Larviciding deployment duration was simulated for 120 and 356 days, allowing for irregular applications, but with fixed efficacy duration of the minimum time step (5 days). The seasonal deployment was described in terms of the number of months during which larviciding was applied per year (beginning, during or end of the rainy season, during the dry season or throughout the year) (Fig. 2).

### Simulation scenarios

The larviciding parameters were explored with three distinct simulation experiments. Larviciding was simulated: (1) for 365 days at maximum application rate throughout the year and constant transmission (no seasonality); (2) for 120 days at varying application rates and coverage at constant transmission throughout the year; and, (3) for 120 days at maximum application rate starting in different months during the year considering four different seasonality profiles. Simulations 1 and 2 were run with 11 coverage levels, three transmission intensities, 18 unique mosquito density dependency parameter combinations, and three stochastic representations, whereas the coverage levels and density parameters were reduced for Simulation 3 to limit simulation size. Simulations were run for a host population of 10,000 people, with a ‘warm-up’ period equivalent to 60 years before the implementation of larviciding. The ‘warm-up’ period led to the defined level of transmission assumed to result from previous interventions not explicitly simulated (Table 1).

**Table 1:** Summary of simulation experiments and varied model parameter

	Simulation 1	Simulation 2	Simulation 3
Setting			
Transmission intensity (ibpa)	3, 10, 90	3, 10, 90	3, 10, 90
Transmission seasonality	–	–	None, medium, high, one peak, two peaks (based on [78])
Intervention deployment			
Coverage	0–1, interval of 0.1	0–1, interval of 0.1	0–1, interval of 0.2
Duration (days)	30, 60, 90, 120, 365, 730, 1095	120	120
Frequency (interval in days)	5**	5, 10, 15, 20, 25, 30, 60, 90	5**
Decay* (days)	5	5	120
Seasonal deployment (month larviciding started)	–	–	1, 2, 3, 4, 5, 6, 7, 8, 9, 10, 11, 12
Mosquito density dependency parameters***			
Female eggs laid	99, 50, 200	99, 50, 200	99
Development survival	0.6, 0,1, 0.9	0.6, 0,1, 0.9	0.6
Development duration (days)	11, 5	11, 5	11

^*^A step function with constant effectiveness for the specific number of days

^**^Minimum time step size in OpenMalaria

^***^ aluation period, either after larviciding was stopped or onBased on OpenMalaria default parameters with upper and lower value for female eggs laid based on [79] and assumed maximum range for survival probability of larvae

### Analysis of simulation outputs

The outputs of the simulations included the number of larvae emerging and surviving to first feeding cycle (mosquito emergence), the number of host-searching mosquitoes, the EIR, and the *Plasmodium falciparum* parasite rate in the human population, assessed during the intervention period, at the end of the intervention period or one year after the intervention period. The impact of larviciding was assessed by comparing the scenarios with larviciding to those without larviciding, defined as the counterfactual. The relative reduction (RR) compared to no larviciding, was calculated per 5-day time step paired by deployment parameters. The mean of the RRs per time step was calculated for the entire duration (*meanRR*). The equations are shown below, where *t* denotes the time step and *n* the total number of time steps, either at the end of the larviciding intervention or one year after intervention start, in the results specified in the figure captions.

$$RR_{t} \, = \,\left( {X\left[ {Counter\,factual_{t} } \right] - X\left[ {larviciding_{t} } \right]/\,X\left[ {Counter\,factual_{t} } \right]} \right)$$

$$mean\,RR\, = \,\frac{1}{n}\sum {\frac{n}{t = 0}RR_{t} }$$

where, X[.] denotes the model outputs (EIR,vector density or prevalence) for either the counterfactual scenario or with larviciding intervention.

The loss in the effectiveness was defined as 1-meanRR calculated at the end of the evaluation period, either after larviciding was stopped or one year after larviciding started. For the prevalence and EIR, the predicted value at the time point (at the end of the evaluation period) was taken, whereas for the vector outcomes the average was taken. The three seeds were averaged and the simulated range among the mosquito population density-dependency parameters was used to obtain uncertainty intervals. Linear regression models were run to quantify the relationship between deployment frequency or coverage and reduction on prevalence.

### Simulation to represent a study site

Additional simulations were run to compare the predicted with the reported impact of larviciding and to establish the relationship between effective coverage and reported operational coverage based on a field study conducted before other vector control interventions were scaled up [6]. In that study, larviciding was applied in Mbita, a rural village in western Kenya, between June 2002 and September 2004 using *B. thuringiensis israelensis* and *B. sphaericus*. The number of treated breeding sites per deployment ranged from 65 to 219 among the 50 applications during the study period. Simulation scenarios intended to represent the study site and informed parameters as reported for the annual baseline transmission intensity, the seasonality, vector species, time of larviciding applications and the larvicide, only varying the coverage. A detailed description of the study is available in the publication by Fillinger et al. [6], and the simulations set-up is included in Additional file 4.

## Results

### Impact of effective coverage and duration of larviciding (Simulation 1)

**Fig. 3:**
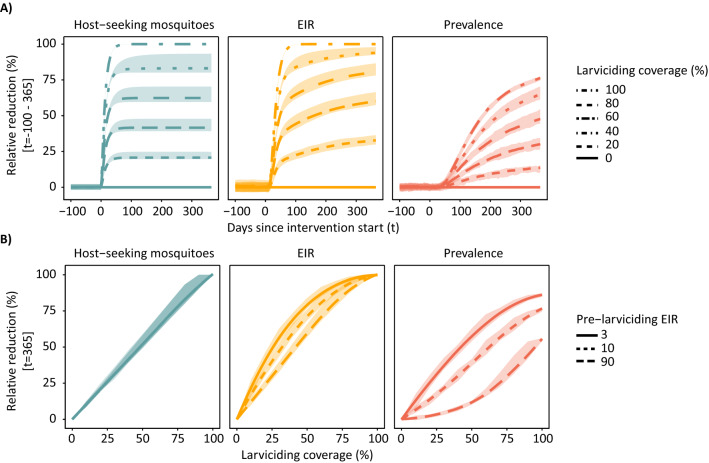
Simulated relative reduction in host-seeking mosquitoes, EIR and prevalence after one year of larviciding. **A** Relative reduction for different coverage levels over time at moderate transmission (EIR = 10 ibpa). **B** Relative reduction by coverage at different transmission intensities after one year. The shaded area indicates the minimum and maximum range among variation in the mosquito density-dependence parameters. The *t* denotes the time in days after intervention start

The impact of larviciding coverage and duration is shown in Fig. 3. After one year of larviciding at 60% coverage, the number of host-seeking mosquitoes was reduced by 62% (range 60–70%), the EIR by 81% (range 78–86%), and the prevalence by 48% (range 45–55%), assuming moderate transmission and no seasonality (Fig. 3A). The number of host-seeking mosquitoes and EIR appeared to reach equilibrium ahead of malaria prevalence rates. The effects on host-seeking mosquitoes started immediately after larviciding but EIR started to decrease after around 10 days and faster than for host-seeking mosquitoes. Malaria parasite prevalence was predicted to decrease around two months after larviciding and had the lowest RRs compared to the other outcomes.

The relationship between effective coverage and reduction in prevalence depended highly on the pre-intervention transmission intensities, with the greatest RRs being in low-transmission settings. An effective coverage above 60% can be considered as high, since it refers to the reduction in all emerging adult mosquitoes, whereas in practice not all breeding sites might be identified, and treatment might not affect all premature stages within a breeding sites equally (Additional file 3). Taking 60% effective coverage as an example, the RR in prevalence at low EIR was around four times higher than at high EIR (meanRR_EIR-3_ = 66% *vs.* meanRR_EIR-90_ = 17%) (Fig. 3B), while at 20% coverage the RR was almost 10 times higher (meanRR_EIR-3_ = 25% vs. meanRR_EIR-90_ = 2.5%). The population density dependency parameters did not substantially influence these relationships, nor the immediate effect of larviciding, however extreme values considerably delayed re-population after high reductions in the mosquito population (Additional File 2: Fig. S2.2 and S2.3). Coverage of larviciding strongly influenced the overall impact of the intervention, except at high transmission intensities (EIR > 90 ibpa) where larviciding was predicted to not have much impact, showing the higher the transmission intensity the lower the impact of the intervention.

### Impact of deployment frequency of larviciding (Simulation 2)

**Fig. 4:**
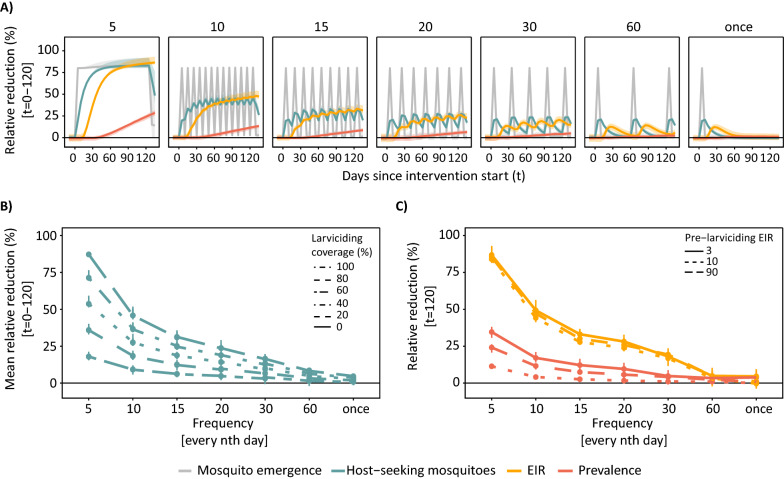
Relative reduction in outcome measures resulting from varying deployment frequencies. **A** Impact of larviciding on the different outcomes measured over time and per deployment frequency (panels) for 80% coverage and in moderate transmission intensity (EIR = 10 ibpa). **B** Mean relative reduction in mosquito emergence during the intervention period by frequency and larviciding coverage irrespective of transmission intensity (averaged for EIR values of 3, 10 and 90 ibpa). **C** Relative reduction in EIR and prevalence at the end of the larviciding deployment period with larviciding at 80% coverage and varying frequency. The *t* denotes the time in days after intervention start. The Figure shows simulation results for a deployment period of 120 days (no seasonality)

The highest impact at any larviciding coverage was achieved at maximum duration of the intervention period (assumed to be 120 days for this specific simulation) (Fig. 4). In practice, this could be achieved through frequent deployments with short-lived larvicides or fewer deployments when using larvicides that have longer residual efficacy. Interrupting the effective coverage by deployment every 10 instead of every 5 days resulted in a loss of effectiveness by 54% (51–56%) for mean host-seeking mosquito density, by 45% (39–49%) in EIR, and by 53% (45–70%) in prevalence. This had assumed coverage of 80%, averaged over the three transmission intensities. For host-seeking mosquitoes, there was high interaction between frequency and coverage with higher loss in impact at high than at low coverage (Fig. 4B). For EIR and prevalence (Fig. 4C), the levels of pre-larviciding transmission intensity influenced the impact of deployment frequency on prevalence but not on EIR. For instance, for a deployment of larviciding at 80% every 10 days (when assuming a short-lived larvicide effective for 5 days), the resulting RR in EIR was predicted at 49% (46–56%) for a pre-intervention EIR of 3 ibpa, 47% (45–53%) for a EIR of 10 ibpa, 44% (41–50%) for a EIR of 90 ibpa. The corresponding RRs in prevalence were 17% (14–21%), 12% (9–13%) and 4% (3–5%) for EIR of 3, 10, and 90 ibpa, respectively.

An increase of 1% in coverage would lead to an average increase of the impact in prevalence by 0.10%, while an additional lag of 5 days between deployments would decrease the RR in prevalence by 2.32% (Fig. 4). To achieve and maintain high impact of larviciding with a short efficacy, the frequency of deployments was more important than the coverage. For instance, an increase in the coverage from 40 to 80% for deployment every 5 days increased the RR in prevalence from 18 to 36%, whereas not even 100% coverage could compromise fewer deployments that leave gaps in effective coverage to achieve the same reduction (Additional file 2: Fig. S2.4).

### Impact of timing on effectiveness of larviciding (Simulation 3)

The timing of larviciding relative to the transmission season substantially influenced the impact on the prevalence. Regardless of seasonality, transmission intensity or coverage, larviciding in the rainy season was most impactful in reducing EIR and prevalence, followed by deployment during the beginning of the rainy season, followed by deployment at the end of the rainy season. For highly seasonal settings, larviciding during the rainy season (for 120 days) was predicted to have a similar impact as deployment all year round, whereas larviciding at the end of the rainy season or during the dry season (for 120 days) had a very low impact. In moderate endemicity setting (EIR = 10 ibpa), larviciding at 80% coverage was predicted to reduce the prevalence after one year by 58% when deployed all year round, by 57% when implemented during the middle of the rain season, by 40% when implemented at the beginning of the rainy season, by 9% at the end of the rainy season and no reduction when deployed at the dry season. The RR in prevalence was on average across the deployment timing 10% higher at lower transmission (EIR = 3 ibpa) and 20% lower at higher transmission (EIR = 90) compared to the reduction simulated for the moderate transmission level (Additional file 2: Fig. S2.9).

**Fig. 5:**
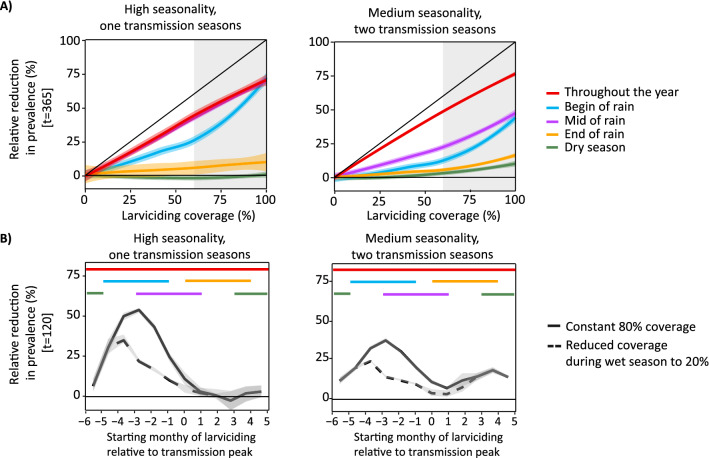
Relative reduction in prevalence due to larviciding compared by seasonal timing at moderate transmission intensity (EIR = 10). **A** Reduction in prevalence after one year with larviciding deployed for one year compared to 120 days at varying seasons. The x-axis shows the effective larviciding coverage, with coverage above 60% shadowed in grey as these coverage levels might be difficult to achieve in practice [54, 56]. The diagonal black line indicates a 1-to-1 relationship. **B** Relative reduction in prevalence at the end of the deployment period (120 days) at moderate transmission for varying deployment starts, relative to the peak in transmission. The t denotes the time in days after intervention start. Mosquito density-dependence parameters were fixed and uncertainty intervals are not shown

The optimal deployment timing for highest impact on prevalence was found to be three months before the peak in transmission, assuming lasting effectiveness until one month after the peak. When coverage of larviciding during the rainy season was reduced to 20% (intended to represent operational challenges to cover all breeding sites during the rainy season), the impact of larviciding, although substantially lower (19% difference in peak meanRR), remained higher when deployed during the dry season, especially at high seasonality with one transmission season. At medium seasonality and two transmission seasons, the optimal deployment timing became less distinct (Fig. 5B). The model predictions therefore suggest that timing the larviciding deployment to the rainy season would be more impactful, even at lower coverage, than achieving high coverage during the other seasons, even when the effective coverage would drop to a coverage of 20%, given the transmission and seasonality scenarios considered in this analysis. Additional seasonal plots are provided in the Additional file 2: Fig. S2.7–10.

### Comparison of deployment factors and post-larviciding resurgence (Simulation 2)

**Fig. 6:**
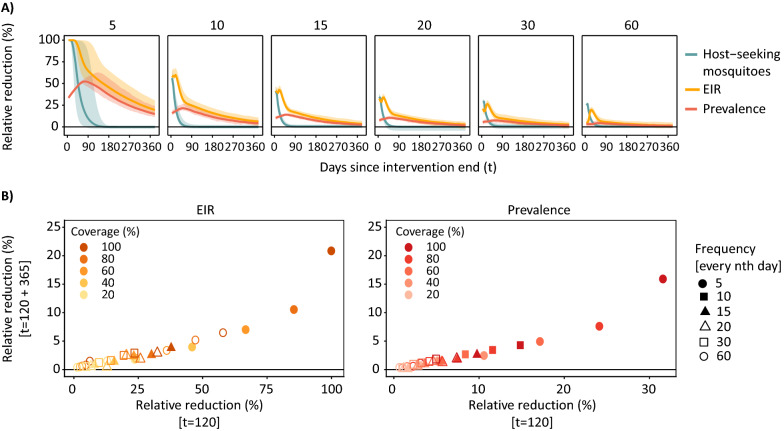
Residual impact in outcome measures by deployment frequencies.** A** Decay of achieved impact on the different outcomes measured over time after stopping larviciding per deployment frequency (panels) for a demonstrative 100% coverage at moderate transmission intensity (EIR = 10 ibpa). **B** Remaining relative reduction in EIR and prevalence by coverage and frequency after one year of intervention stop compared to at the end of the intervention period. Note the relative reduction in prevalence is selected at the specified time steps while the prevalence reaches a maximum around 30–60 days after the intervention period of 120 days (Fig. A2.6). Results shown for a deployment period of 120 days (no seasonality) and moderate transmission intensity, with uncertainty intervals and error bars corresponding to simulated range of mosquito density-dependence parameters

After the end of an assumed intervention period of 120 days, the number of host-seeking mosquitoes resurged immediately to pre-larviciding levels whereas the EIR, after an initial drop, resurged at a slower rate and the prevalence resurged at the slowest rate after a delay of 25–30 days (Additional file 2: Fig. S2.5). A year after the end of the intervention period, the EIR and prevalence did not return fully to pre-larviciding levels, depending on the deployment factors and achieved impact. The higher the maximum reduction during the intervention period (as a product between coverage and frequency), the slower the resurgence to initial levels (Fig. 6). The reduction in EIR and prevalence that remained after the intervention period also varied depending on when larviciding was applied as well as the seasonal pattern (Additional file 2: Fig. S2.6).

### Re-simulated larviciding study

In the selected field study in Mbita, western Kenya, larviciding was reported to reduce the larval density by 95%, the adult density by 92%, and the EIR from 9 to 0.8 ibpa during the two-year intervention period compared to the pre-and post-intervention period [6]. Larviciding was simulated with the same number of deployments as reported (on average every 11 days when using *B. thuringiensis israelensis*, every 22 days when using *B. sphaericus* (Additional file 4: Table S4.1)) with a range of larviciding coverage between zero and 100%, using an input EIR of 10 ibpa. At an assumed effective coverage of > 90%, the maximum reduction in larval density was around 77%, in adult density 67%, and in EIR 40%. The reported high reductions in adult density and EIR of > 90% could only be simulated with assumed constant effectiveness and unrealistically high coverage (predicted to reduce the larval density by 94%, the adult density by 93% and the EIR from 10 to 1.37). The results are shown in Additional file 4.

## Discussion

This modelling study investigated the impact of larviciding deployment strategies varying by coverage, duration, application frequency, and seasonal timing, for three transmission intensities (3, 10 or 90 ibpa), and five seasonality patterns, assuming homogeneous vector population similar to *An. gambiae*. Overall, larviciding impacted the prevalence at a slower rate than the number of host-seeking mosquitoes and transmission intensity, while reduction in prevalence remained beyond the intervention period. The effective coverage during the intervention period, as a result of the efficacy duration of the larvicide, frequency and emergence reduction (effective coverage per single application), highly influenced the impact of larviciding. To ensure high impact, the product of the deployment factors need to be high, with regular deployments, tailored at the efficacy duration of the larvicide being more important than the effective coverage (number of emerging mosquitoes killed at each single round of larviciding) even at high coverage, when assuming short-lived larvicides. In highly seasonal settings, the deployment during the rainy season was predicted to have the highest impact on EIR and prevalence even at much lower coverage than during the dry season, and dry season larviciding had negligible impact. Larviciding at lower compared to high transmission intensity was further predicted to have a higher epidemiological impact with greater and longer lasting RR in the prevalence. This difference could be attributable to the differences in mosquito densities and faster rate of re-establishment at high transmission after lavicide decay to be effective (Additional file 2: Fig. S2.5).

Field observations [4, 9] and simulations agree that larviciding reduces the number of emerging mosquitoes for the duration of the killing effect and that the vector population re-establishes immediately afterwards [4, 37, 80]. In field studies, the time to reduce numbers of host-seeking mosquitoes varies between immediate impact and to lag times of two to three weeks [4, 37, 80], with reductions in host-seeking mosquitoes ranging from very low to almost as high as the reduction in observed larval density [6, 17, 40]. The simulations showed that prevalence is affected at a slower rate than the mosquitoes and transmission intensity and did not reach an equilibrium after one year of constant larviciding. This relates to the important role of duration of infection and parasite reservoirs in humans. It requires more time to clear infections in the human population by only reducing the mosquito population, whereas the reduction will also depend on malaria case management, which was not included in the simulations. However, this finding indicates that longer follow-up times would be required in field studies to capture impact of larviciding on prevalence with follow-up times varying depending on the seasonality.

Shorter intervals between deployments to reduce gaps in effective coverage over time were predicted to increase the average impact and reduce fluctuations in outcomes, as observed in two studies in Kenya [6, 81]. In practice, the required deployment frequency depends on the emergence rate of new breeding sites and the persistence of the specific active agent [5, 7, 17, 42, 82]. Notably, some programmes focus on treating only productive breeding sites [10, 59], a strategy considered cost-effective in a rural district in Burkina Faso [25]. Concentrating efforts on peri-domiciliary breeding sites has also been advocated [83]. The appropriate deployment strategy to achieve high coverage of larviciding, or LSM in general, will further depend on dispersal of breeding sites and total land area to cover, surface area and quantity of breeding sites as well as their proximity to houses.

Larviciding is currently recommended by the WHO to be deployed in areas or seasons where breeding sites are fixed, few and findable, commonly associated with the dry season or urban areas [1, 2], however, the simulation results suggest that larviciding in the dry season would have limited impact in seasonal settings. The results further suggest that the additional benefit of larviciding throughout the year would be marginal in highly seasonal settings. The greatest impact on prevalence was predicted when implementation preceded the peak in transmission, hence averting seasonal increases in host-seeking mosquito density. However, rainy season larviciding is more challenging, in particular because of proliferation of breeding sites and dilution of larvicide [17, 29, 33, 81], while on the other hand emergence rates might be reduced when larvae are flushed away by very high rainfall [18, 81, 84]. The trade-off between achieving high coverage (often described as more feasible in the dry season or arid areas [1, 85]) and the epidemiological impact associated with a given coverage (in simulation estimated higher in the rainy season) must play out differently in diverse environments and might well account for some of the variation in seasonal patterns of impact observed in the field. In this modelling study, the operational challenge was attempted to reflect lowering the coverage during the rainy season while keeping the coverage during the dry season high, which did not change the recommended timing for larviciding unless coverage dropped to less than 20% of emerging mosquitoes killed. Alternative approaches to adjust for operational challenges would include simulating shorter effectiveness [29] or more frequent deployments [81] in the rainy season, presumably with similar implications. The results apply for settings with low vector densities and little to no transmission during the dry season, and where peak in transmission follows with one month lag after peak in rainfall.

The model results suggest that the relative impact would be greater at low than at high transmission in which high coverage would be needed. Nevertheless, larviciding has been successfully deployed in moderate to high transmission areas in several studies [27, 37, 86]. One study in particular showed that larviciding could be implemented at high transmission in highly seasonal areas with findable breeding sites [86]. Reduction in prevalence has rarely been studied in larviciding field studies [9] although one study reported a reduction of more than 70% [26]. Based on the simulations, such high reductions would only be achievable at very high coverage and long duration of effective larviciding, either with more frequent deployments or longer residual activity (e.g., longer than 120 days), and seems unlikely to be achieved with larviciding alone as reported in the study.

In most instances, larviciding, recommended as a supplementary intervention [1], will be deployed alongside ITNs or IRS, to reduce transmission and create a context where larviciding is more effective. While interactions were not explicitly modelled, implicitly synergistic effects with these interventions were assumed by simulating low pre-larviciding transmission intensity. This assumption is supported by the higher impact at low transmission seen in the predictions. For the same reason, synergies with chemotherapeutic interventions can also be anticipated [87]. Further synergies are likely where there is insecticide or drug resistance because larvicides have different biochemistry and act independently of host-seeking and resting behaviour of adult mosquitoes [14], and they can address transmission that is refractory to the core interventions. In practice, larviciding might also be combined with other LSM approaches [1, 88, 89] that together reduce the adult mosquito emergence in an area.

Although the measurement of coverage is critical for predicting the impact of a larviciding programme, there is no standardization of operational coverage measures. These have been variously defined as the number of treated breeding sites out of the total identified, the proportion of the surface area of water bodies that are treated, or even the proportion of larvae covered by larvicide out of all larvae within a breeding site (Additional file 3). Targeting specific areas (or selection of sub-sets of breeding sites or other criteria) reduce the denominators in such calculations. All these measures of coverage are challenging to estimate [8, 12, 15, 90], especially since the proportion of breeding sites identified varies in each setting and over time. Regardless of the suitability of the local settings, the effectiveness also depends on the performance of field staff, community engagement and supervision [91, 92].

The simulations of larviciding in Mbita, western Kenya, attempted to calibrate the model to allow for these factors. The results emphasize the difficulty of correctly reproducing the impact of larviciding, and on estimating coverage levels that would be feasible, despite accounting for details of deployment. In the simulations, the vector population immediately increased between the larvicide applications, whereas in the field measurements adult densities remained relatively low [6]. Hence, the low levels in host-seeking mosquito density maintained throughout the intervention period of two years could only be reproduced with constant high effective coverage. It could be that the sampling under-represented the true adult mosquito density in the community or that the simulated re-treatment intervals underestimated the effectiveness in practice. Another reason could be additional use of ITNs or other factors not accounted for in the simulations that lowered the transmission throughout the study period.

In contrast to the homogeneous vector populations in the simulations, multiple vector species are usually present in the field, and some of this variation in outcomes result from environmental and ecological factors that cannot be captured in the model. For instance, larviciding of rice fields has been found to be impractical in The Gambia, due to low accessibility [40], but was feasible in Tanzania and Rwanda [36, 93]. One study in Kenya found positive effects of dry season implementation on mosquito density and clinical malaria using long-lasting larvicides [17]. Another study in western Kenya reported higher effectiveness during the rainy season, using short-lived larvicides [94]. For instance, while a high number of breeding sites existed throughout the dry season in an urban setting (Dar es Salaam) [11], they substantially varied by season in the rural village setting in Mbita, western Kenya [6]. Hence, field operations should always consider local climate, breeding site permanence based on water sources and characteristics, dominant vector species, available resources, and engagement of the community [95]. The diversity of operational implementation and outcomes highlights the need for more setting-specific guidelines for larviciding to differentiate between strategies for different localities. For instance, in Tanzania, the national malaria strategic plan includes larviciding the whole country [96], but heterogeneities in malaria epidemiology and environmental factors represent a huge challenge for planning appropriate large-scale strategies [97, 98] and implementation will require a thorough assessment of the context at local level.

## Conclusion

In seasonal transmission settings, larviciding was predicted to be most impactful if done before and during the peak in vector density; in many settings this corresponded to the rainy season instead of during the dry season as currently recommended by WHO. Some deployment parameters, including coverage, are difficult to determine accurately in reality *versus* in a model. Field studies find substantial variation in outcomes that appears to stem from diversity in eco-environmental settings, vector biology and in operational strategies, and are often difficult to relate to model predictions. To make model-based impact predictions that can be compared between areas, the different deployment strategies and coverage should be calibrated against effects on densities of host-seeking vectors and prevalence in humans. Such calibration would enable models to become a practical tool to support malaria control programmes in developing operational strategies for larviciding that account for diversity of context.

## Supplementary Information

**Additional file 1:** A simple periodically forced difference equation model for mosquito population dynamics.

**Additional file 2:** Additional result figures.

**Additional file 3:** Flowchart from operational to effective larviciding coverage.

**Additional file 4:** Re-simulated larviciding study in Mbita, western Kenya between 2002 and 2006.

## Data Availability

The analysis code is available on GitHub at https://github.com/ManuelaRunge/om_larviciding_tza. The simulation output is available from Zenodo at https://doi.org/10.5281/zenodo.5033187.

## Abbreviations

DDT: Dichlorodiphenyltrichloroethane
EIR: Entomological inoculation rate
ibpa: Infectious bites per person per annum
IRS: Indoor residual spraying
ITN: Insecticide-treated bed nets
LSM: Larval source management
RR: Relative reduction
*t*: Time in days
