# Correction to: Evaluation of different deployment strategies for larviciding to control malaria: a simulation study

**DOI:** 10.1186/s12936-021-03882-0

**Published:** 2021-08-26

**Authors:** Manuela Runge, Salum Mapua, Ismail Nambunga, Thomas A. Smith, Nakul Chitnis, Fredros Okumu, Emilie Pothin

**Affiliations:** 1 Swiss Tropical and Public Health Institute, Basel, Switzerland; 2 University of Basel, Basel, Switzerland; 3 Environmental Health and Ecological Sciences Department, Ifakara Health Institute, Ifakara, Tanzania; 4 Clinton Health Access Initiative, Boston, USA

## Correction to: Malar J (2021) 20:324 https://doi.org/10.1186/s12936-021–03854-4

Following publication of the original article [1], it was brought to the authors’ attention that a word was missing from the first sentence of the Abstract, with the result that the meaning was altered.

This first sentence used to read "Larviciding against malaria vectors in Africa has been limited to indoor residual spraying and insecticide treated nets but is increasingly being considered by some countries as a complementary strategy".

However, the sentence should read as "Larviciding against malaria vectors in Africa has been limited compared to indoor residual spraying and insecticide treated nets but is increasingly being considered by some countries as a complementary strategy." (With the inclusion of 'compared to' after 'has been limited').

The original article has since been updated to correct this.
