# Supplementary material for: Evaluation of different deployment strategies for larviciding to control malaria: a simulation study
Source: Malar J. 2021 Jul 27;20:324. doi: 10.1186/s12936-021-03854-4 (PMC8314573; doi:10.1186/s12936-021-03854-4)
Supplement: Supplementary file 1 — Additional file 1: A simple periodically forced difference equation model for mosquito population dynamics. [file 12936_2021_3854_MOESM1_ESM.docx]

**Additional File 1: VectorModel**^[[1]](#footnote-1)^

The previous developed model for malaria in mosquitoes did not include the dependence of the emergence rate of new mosquitoes on the number of eggs laid [1]. Hence, it did not include the nonlinear effects of adultcides on reducing the population size. To include this effect and, also, to better model the effects of larval control a model for mosquito population dynamics was developed. Most models of mosquito population dynamics have focussed on Aedes mosquitoes and have used ordinary differential equations (ODEs) [2], delay differential equations (DDEs) [3–5], or stochastic individual based models [6–8]. Dye [4] and Yakob et al. [9] developed difference equation models but only for successive generations. However, to link with the existing mosquito model in OpenMalaria, a model for population dynamics with overlapping generations and a discrete time step of one day is needed. It is assumed that the dynamics of the population are regulated by a periodically-varying larval carrying capacity. Further, a simple model where only one juvenile stage is simulated is assumed.

**Table SI-1.1: Model parameters**

| **Parameter** | **Description** |
| --- | --- |
| $T_{p}$ | Period of the system. This period is usually set to 365 days when using a daily timestep.  Dimension: Time.  $T_{p}\mathbb{\in N}$ [1] |
| $\tau$ | Duration of resting period of mosquitoes.  Dimension: Time.  $\tau\in\mathbb{N}$ [1] |
| $\theta_{j}$ | Duration of the juvenile stage.  Dimension: Time.  $\theta_{j}\mathbb{\in N}$ |
| $b$ | Number of female eggs laid by one female mosquito per ovipost.  Dimensionless.  $b>1$ |
| $\rho$ | Survival probability of a mosquito from egg to emergence in the absence of density dependent mortality. |
| $\gamma(t)$ | Resource availability at time $t$.  Dimension 1/Animals. $\gamma\left( t \right)>0. \gamma\left( t+T_{p} \right)= \gamma\left( t \right) \forall t\mathbb{\in N}$ |

In the model of malaria mosquitoes, [1], the population of adult host-seeking mosquitoes was determined by,

$x\left( t \right)=g\left( x\left( t-1 \right), x\left( t-\tau\right),t \right)+ E(t)$ (1)

**Table SI-1.2: Derived parameters**

| **Parameter** | **Description** |
| --- | --- |
| $P_{df}$ | Probability of finding a host and surviving the feeding cycle. Dimensionless.  $0<P_{df}<1$ [1] |
| $E(t)$ | Emergence rate of adult mosquitoes at time $t$.  Dimension: Animals.  $E\left( t \right)>0. E\left( t+ T_{p} \right)= E\left( t \right) \forall t\mathbb{\in N}$ |

Where $x\left( t \right)$ is the population of adult host-seeking mosquitoes at time $t$, $E(t)$ is a fixed periodic sequence of emerging mosquitoes, and $g\left( x\left( t-1 \right), x\left( t-\tau\right),t \right)$ determines the survival of adult mosquitoes. Here, we extend (1) to allow the emergence rate to depend on the adult population using a Beverton-Holt model,

$x\left( t \right)=g\left( x\left( t-1 \right), x\left( t-\tau\right),t \right)+ \frac{\rho y\left( t-\theta_{j} \right)}{1+\gamma\left( t-\theta_{j} \right)y\left( t-\theta_{j} \right)},$ (2a)

$y\left( t \right)=bP_{df}x(t-\tau)$ (2b)

Where $y\left( t \right)$ is the population of juvenile stages at time $t$. To initialize the system, we assume that the system (2) has a locally asymptotically stable periodic orbit that is has reached. In that case the estimated periodic emergence rate is

$E\left( t \right)= \frac{\rho y\left( t-\theta_{j} \right)}{1+\gamma\left( t-\theta_{j} \right)y\left( t-\theta_{j} \right)}$ (3)

$= \frac{\rho bP_{df}x\left( t-\theta_{j}-\tau\right)}{1+\gamma\left( t-\theta_{j} \right)bP_{df}x\left( t-\theta_{j}-\tau\right)}$ (4)

Solving (4) for $\gamma(t)$ provides^[[2]](#footnote-2)^,

$\gamma\left( t-\theta_{j} \right)=\frac{\rho bP_{df}x\left( t-\theta_{j}-\tau\right)-E\left( t \right)}{E\left( t \right)bP_{df}x\left( t-\theta_{j}-\tau\right)}$ (5)

To simulate larviciding, we can use

$x\left( t \right)=g\left( x\left( t-1 \right), x\left( t-\tau\right),t \right)+(1-c) \frac{\rho y\left( t-\theta_{j} \right)}{1+\gamma\left( t-\theta_{j} \right)y\left( t-\theta_{j} \right)}$ (6a)

$y\left( t \right)=bP_{df}x(t-\tau)$ (6b)

Where $c$ is the proportional coverage of breeding sites treated with larvicides with$0<c<1$.

# References

1. Otero M, Solari HG, Schweigmann N. A Stochastic Population Dynamics Model for Aedes Aegypti: Formulation and Application to a City with Temperate Climate. Bull Math Biol. 2006;68:1945–74.

2. Cooke K, van den Driessche P, Zou X. Interaction of maturation delay and nonlinear birth in population and epidemic models. J Math Biol. 1999;39:332–52.

3. Dye C. Models for the Population Dynamics of the Yellow Fever Mosquito, Aedes aegypti. J Anim Ecol. 1984;53:247–68.

4. Hancock PA, Godfray HCJ. Application of the lumped age-class technique to studying the dynamics of malaria-mosquito-human interactions. Malar J. 2007;6:98.

5. Ahumada JA, Laoointe D, Samuel MD. Modeling the Population Dynamics of Culex quinquefasciatus (Diptera: Culicidae), along an Elevational Gradient in Hawaii. J Med Entomol. 2004;41:1157–70.

6. Depinay J-MO, Mbogo CM, Killeen G, Knols B, Beier J, Carlson J, et al. A simulation model of African Anopheles ecology and population dynamics for the analysis of malaria transmission. Malar J. 2004;3:29.

7. Focks DA, Haile DG, Daniels E, Mount GA. Dynamic Life Table Model for Aedes aegypti (Diptera: Culicidae): Analysis of the Literature and Model Development. J Med Entomol. 1993;30:1003–17.

8. Yakob L, Alphey L, Bonsall MB. Aedes aegypti control: the concomitant role of competition, space and transgenic technologies. J Appl Ecol. 2008;45:1258–65.

9. Chitnis N, Hardy D, Smith T. A Periodically-Forced Mathematical Model for the Seasonal Dynamics of Malaria in Mosquitoes. Bull Math Biol. 2012;74:1098–124.

1. This report is also available online at <https://github.com/SwissTPH/openmalaria/wiki/ModelMosqPopDynamics> [↑](#footnote-ref-1)
2. We again need to ensure that (t) > 0: I assume this will always be the case but there should be a check for it.

   Also, since (t), x(t), and E(t) are periodic sequences, it is OK to solve both forwards and backwards in time. [↑](#footnote-ref-2)
