# Supplementary material for: Evaluation of different deployment strategies for larviciding to control malaria: a simulation study
Source: Malar J. 2021 Jul 27;20:324. doi: 10.1186/s12936-021-03854-4 (PMC8314573; doi:10.1186/s12936-021-03854-4)
Supplement: Supplementary file 2 — Additional file 2: Additional result figures. [file 12936_2021_3854_MOESM2_ESM.docx]

Additional File 2: Additional result figures

Content

[Relationship among outcome measures in absence of larviciding 2](#_Toc72130092)

[Larviciding in absence of seasonality 3](#_Toc72130093)

[Influence of mosquito density-dependence parameters 3](#_Toc72130094)

[Larviciding frequency versus coverage 5](#_Toc72130095)

[Post-larviciding resurgence in predicted outcome measures 5](#_Toc72130096)

[Larviciding and seasonality 7](#_Toc72130097)

[Seasonality and timing of larviciding 7](#_Toc72130098)

[Coverage thresholds and seasonal timing 9](#_Toc72130099)

# Relationship among outcome measures in absence of larviciding

The lag time between peak density of emerging mosquitoes and peak density in host-seeking mosquito was 10 days, followed by 40 days until peak in EIR and 55 days until peak in prevalence. . Relative to peak in density of emerging mosquitoes, the peak in EIR followed after 50 days (1.6 months) and the peak in prevalence after 105 days (3.5 months) (Fig. A2.1A). The incidence (number of new cases) showed the same trend and peak as the simulated EIR (Fig. A2.1B). The average prevalence is 23% for an annual EIR of 3 ibpa, 40% for an annual EIR of 10 ibpa, and 50% for an annual EIR of 90 ibpa, the seasonal variation is shown in Fig A2.1C. More information available in the OpenMalaria wiki^[[1]](#footnote-1)^.


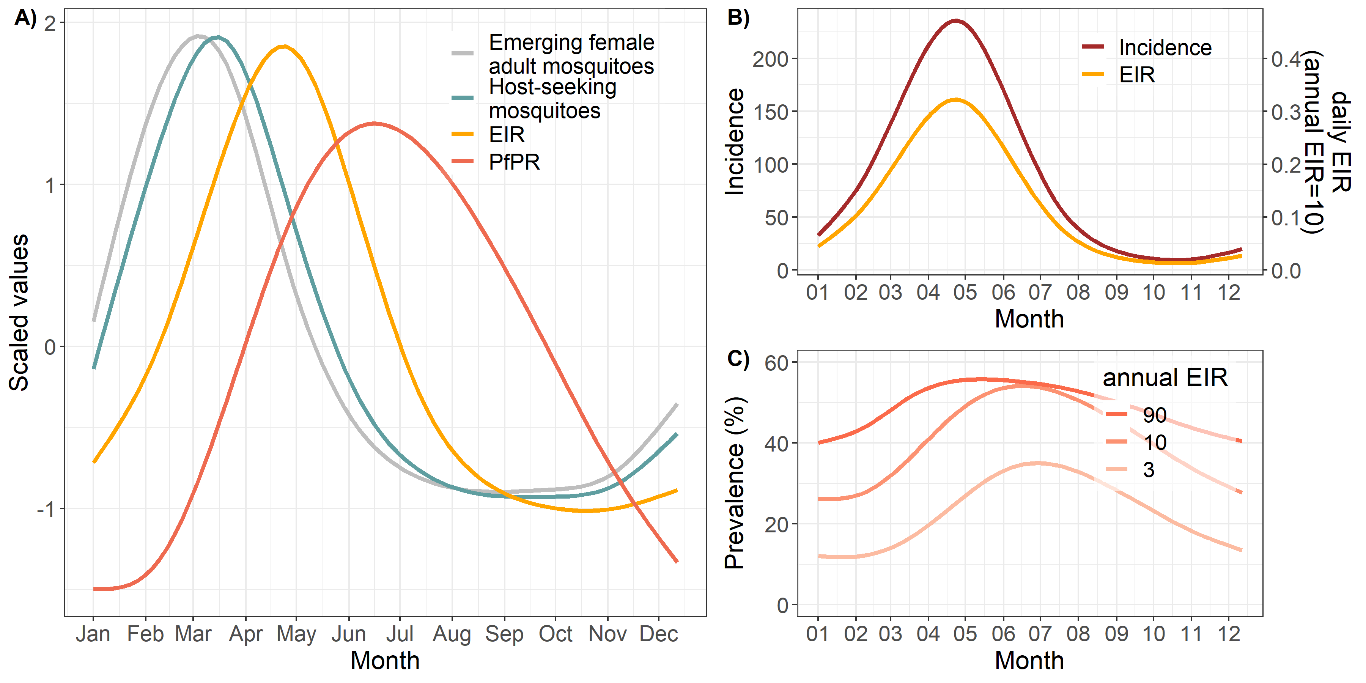


Fig. S2.1: Simulated outcome measures for one year in absence of larviciding for a high seasonaliy setting. A) Comparison of seasonality and delay between mosquito emergence, host seeking mosquito density, EIR and prevalence (values on relative scale). B) Comparison of incidence and EIR over time for an annual EIR of 10. C) Predicted prevalence over time by annual EIR.

# Larviciding in absence of seasonality

## Influence of mosquito density-dependence parameters

Larviciding was simulated at fours coverage levels (20%, 40%, 80%,100%) that stayed constant for 60 days. In addition, three parameters that describe the mosquito density-dependence were varied, the survival probability, the number of eggs laid and the development duration. The range for the survival probability were selected to explore the whole range regardless of biological feasibility whereas for the other two parameters the values were selected based on biological reported development durations for *Anopheles* mosquitoes^[[2]](#footnote-2)^. The model operates on a five day timestep.

On average, the reduction in emerging adult mosquitoes per population over time corresponded to the larviciding coverage, with small variations depending on the mosquito density-dependence parameters. At low coverages (<40%), the initial reduction in emergence was lower for specific mosquito density-dependence parameter combinations, while the emergence reduction increased over time at high coverage (>80%). At high coverage, some combinations of the mosquito density-dependence parameters prolonged the reestablishment of the vector population over time. The parameters affected the rate at which the mosquito population reestablished especially when the population was completely depleted (100% coverage), whith slower reestablishment when the survival probability and numbers of female eggs laid per oviposting were low.The level to which the mosquito population reestablished was similar to the pre-larviciding levelwith few exceptions when the re-establishment rate was slower (Fig. S2.2).

The survival probability was predicted to have the strongest impact on the reestablishment rate and the development durations was predicted to have the lowest impact on the reestablishment rate. When assessing each parameter separately, they did not change the mean predictions substantially, except for the very low survival probability of 0.1%, with wide ranges in the predictions suggesting high interaction among the parameters (Fig. S2.3).


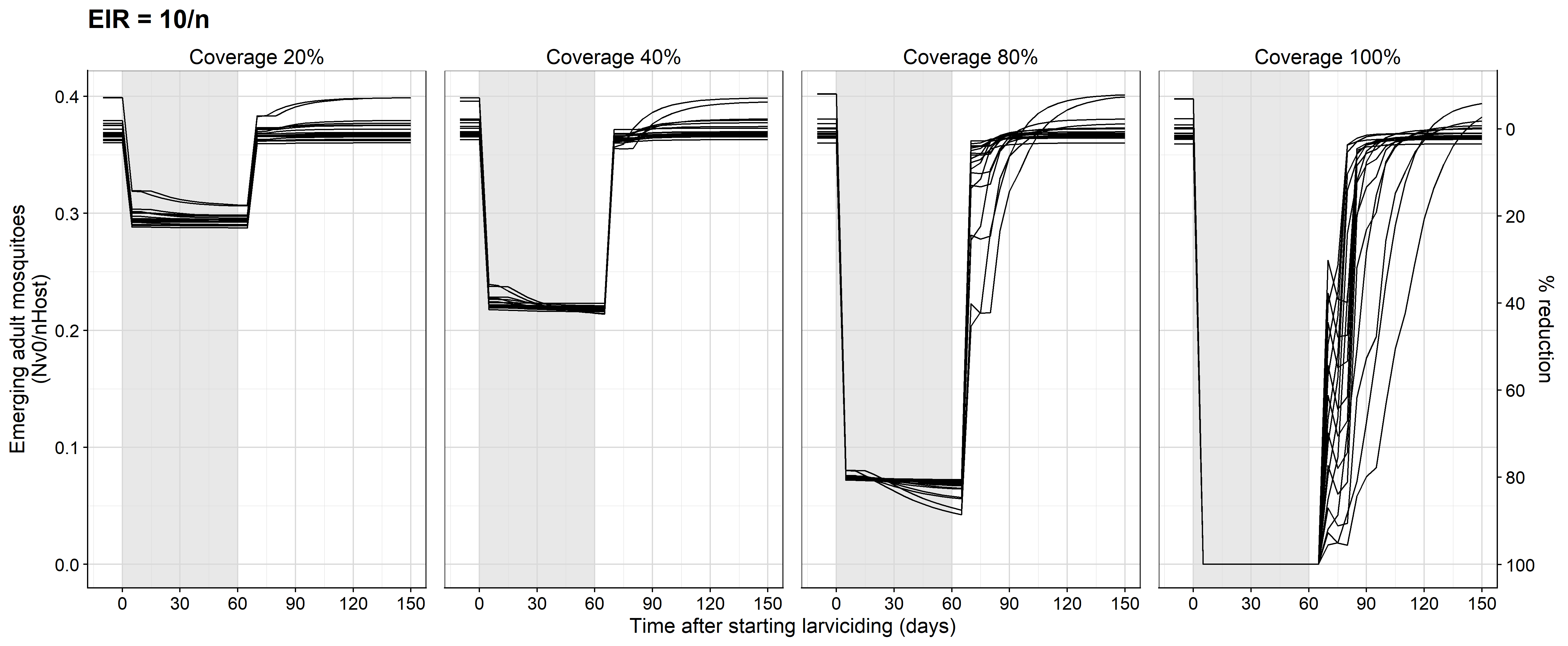


Fig. S2.2: Simulated **impact of** constant larviciding for 60 days **at four different coverage levels on vector density.**

Each line corresponds to a unique set of mosquito density-dependence parameters as shown in Fig S2.3. The figure was identical across the simulated transmission intensities (EIR 3, 10 and 90 ibpa) and this figure shows the predictions at moderate transmission intensity (EIR=10 ibpa). The corresponding emerging adult mosquito to host ratios were 0.15 per 10000 humans at 3 ibpa, 0.37 at 10 ibpa, and 2.7 at 90 ibpa. The model operates at minimum timesteos of 5 days.


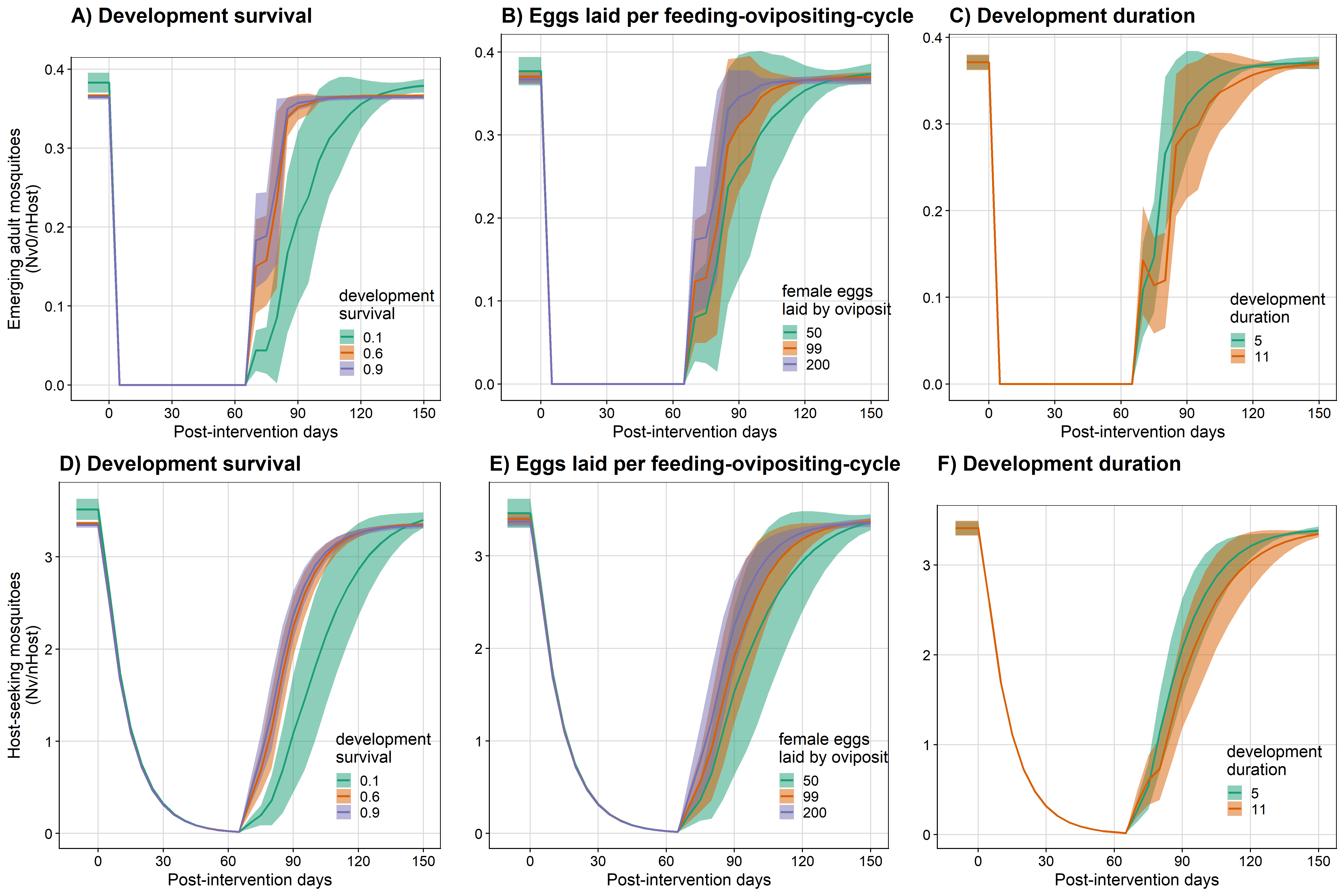


Fig. S2.3: Influence of population density parameters vector populations with constant larviciding for 60 days at 100% coverage.

**A-C)** Emerging adult mosquitoes. **D-F)** Host-seeking mosquitoes. Each panel in a row shows one of the mosquito density-dependence parameter values (colors) while the other two mosquito density-dependence parameters are averaged

## Larviciding frequency versus coverage

The relative reduction in prevalence depending on larviciding coverage and deployment frequency was assessed at the end of the intervention period (120 days) and by transmission intensity as well as for two different decay functions (step decay versus exponential decay). When the deployment frequency or the coverage were reduced by half (e.g. deployments every five to every ten days, coverage from 80 to 40%), the predicted effectiveness was approximately reduced by half as well, assuming a step decay after five days. Overall, fewer deployments led to higher loss in effectiveness than lower coverage. With longer breaks between deployments, the decay rate of the larvicide was predicted to have less influence on the impact. The predicted relationship between frequency and coverage was the same for the three transmission intensities included in the analysis, whereas the maximum reduction was 2.5 times lower at high transmission compared to low transmission (Fig. S2.4).


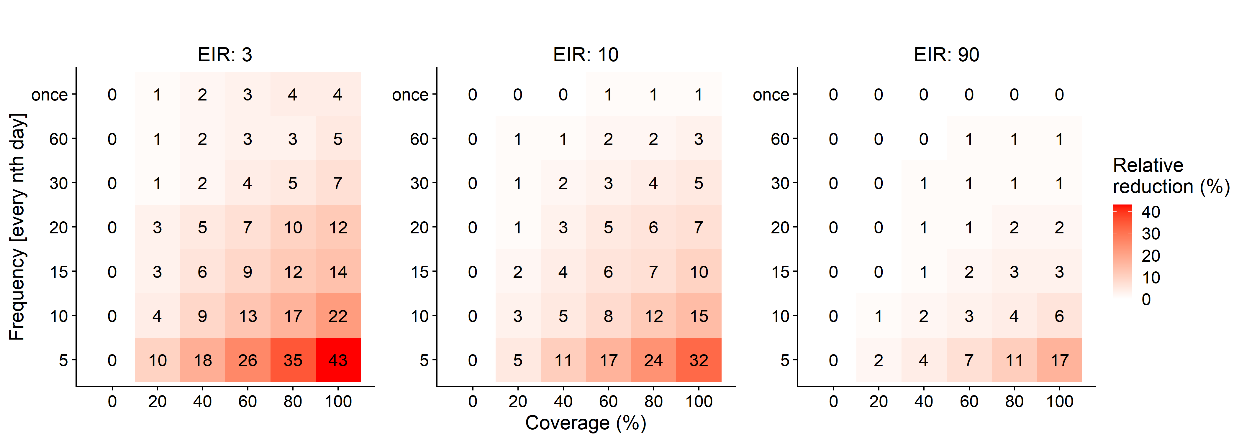


Fig. S2.4: Relative reduction in prevalence by frequency versus coverage at the end of the intervention period (120 days).

The plot shows the relative reduction for an effectiveness decay of 100% for 5 days (1 timestep in OpenMalaria) and reaching 0% directly after (timestep 2).

## Post-larviciding resurgence in predicted outcome measures

The re-surgence in the outcome measures after stopping larviciding depending on the deployment frequency during the intervention period is shown in Fig S2.5. The predicted rebound was similar for all deployment scenarios but very different for the three outcome measures included in the analysis. The reduction in prevalence reached a peak that was much lower as for EIR and adult mosquito density, and the resurgence to pre-larviciding levels was predicted to also take much longer (Fig S2.5). For prevalence, the resurgence was slower at low compared to high transmission intensity (Fig S2.6.)


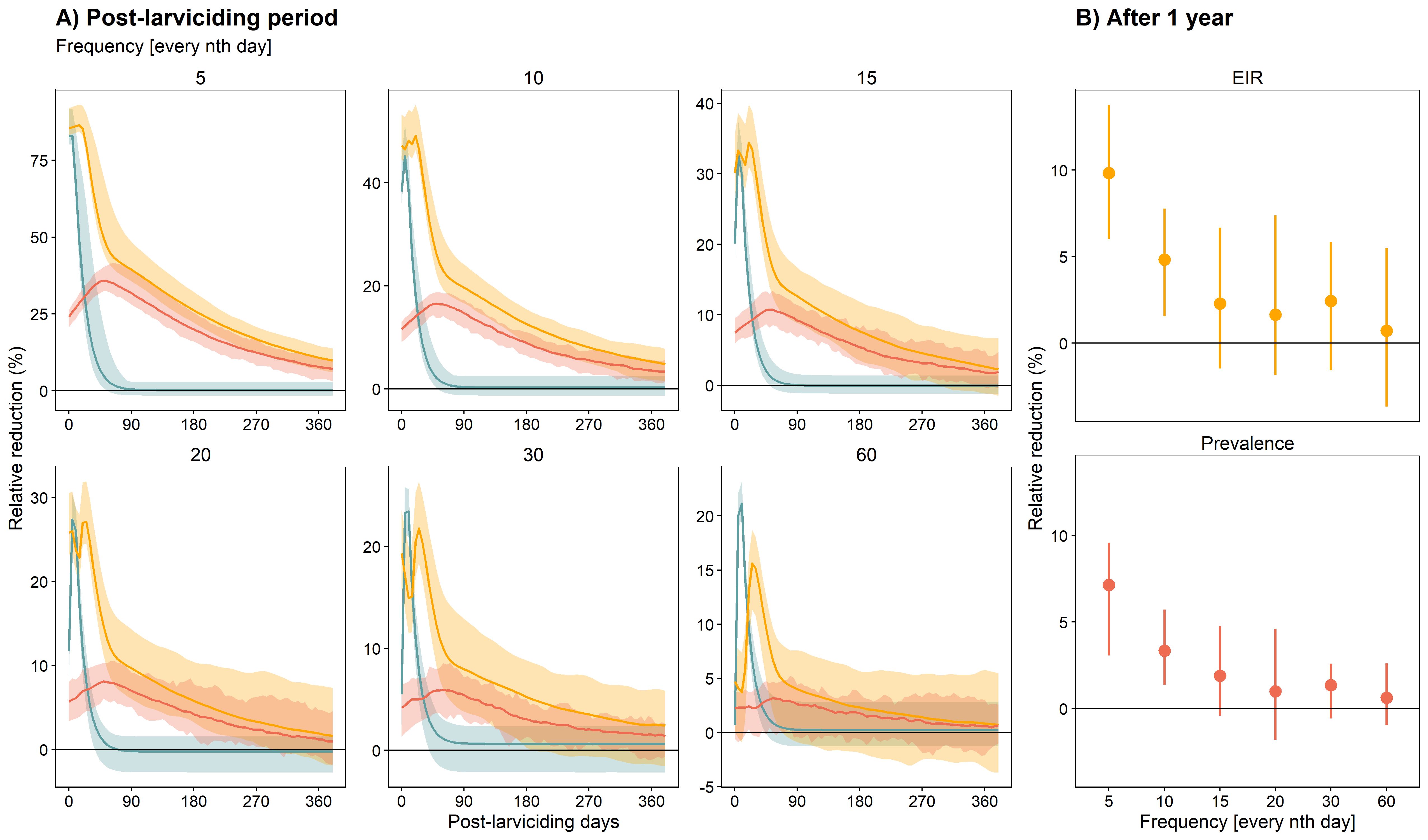


**Fig. S2.5: Varying larviciding deployment and impact on vector density, EIR and prevalence over time with 80% coverage at moderate transmission intensity (EIR = 10 ibpa).**

**A)** Predicted relative reduction compared to no larviciding during the post-larviciding period. **B)** Predicted relative reduction after one year.The uncertainty interval and the errorbars show the range around the mosquito density-dependence parameters (development survival, number of female eggs laid per ovipositing, development duration).

The relative reduction in prevalence showed a lag time of 25-30 days after start of larviciding and 30-60 days after end of larviciding, depending on coverage (Fig S2.6).


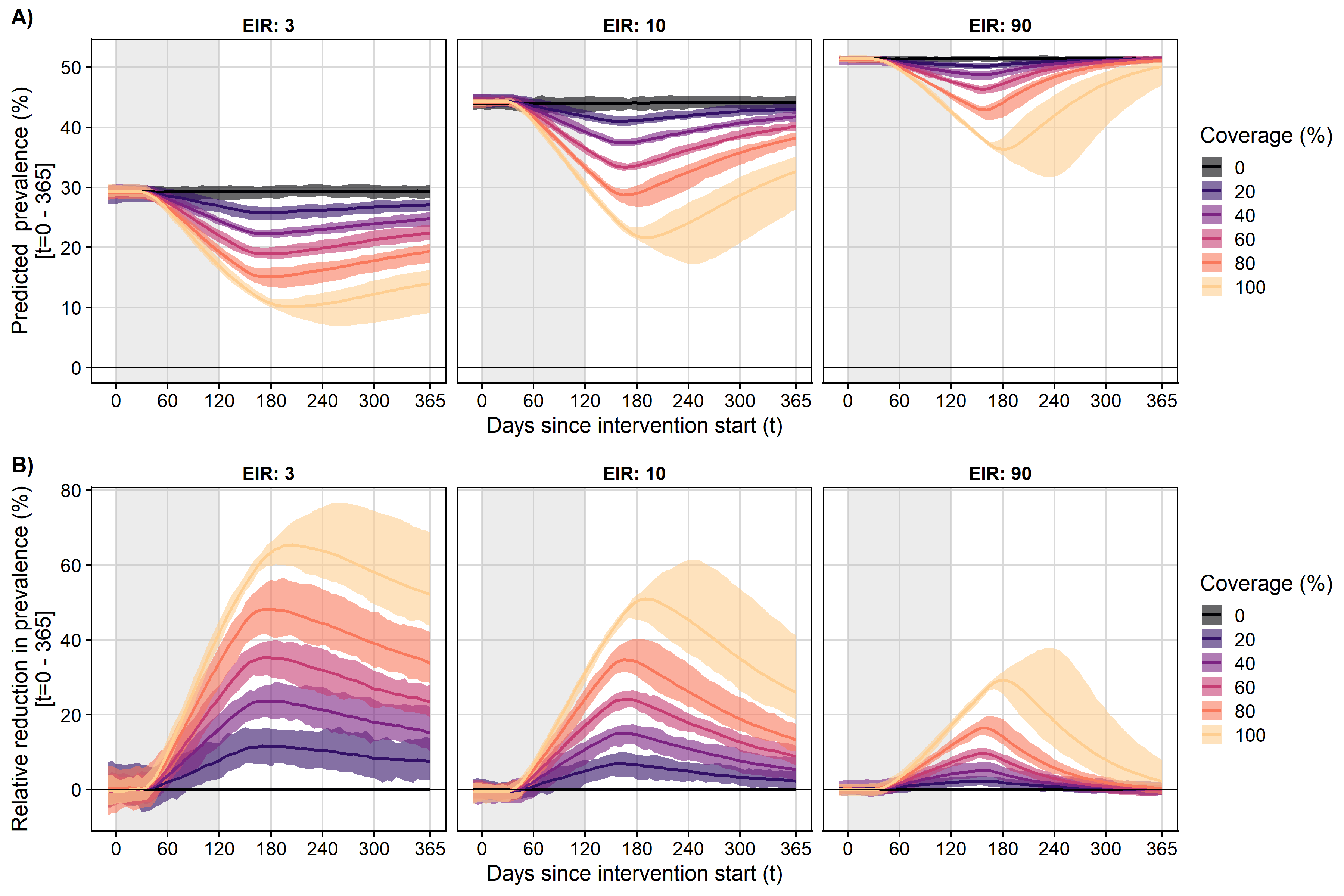


Fig. S2.6: **Impact on prevalence over time** with constant l**arviciding deployment for 120 days at different coverage levels and varying transmission intensity.**

**A)** Predicted prevalence over time. **B)** Relative reduction compared to no larviciding. The grey shaded area indicates the time larviciding was deployed assuming constant effectiveness. The uncertainty intervals show the range around the mosquito density-dependence parameters (development survival, number of female eggs laid per ovipositing, development duration).

# Larviciding and seasonality

## Seasonality and timing of larviciding

The highest reduction was predicted for larviciding starting around three months before the peak in transmission with the assumption of a fixed effectiveness duration of 120 days, lasting until one months after the peak. (Fig. S2.7). Regardless of seasonality, transmission intensity or larviciding coverage, deployment of larviciding in the wet season was the most impactful timing, followed by deployment during the beginning of the rain, then the end of the rainy season. For high seasonal settings, deployment of larviciding during the rainy season only or throughout the whole year would have a similar expected impact. During the dry season, the relative impact of larviciding was equally low across all transmission intensities. The modelling results further suggest that at very high transmission intensity with perinnial transmission (no seasonality) larviciding would not have any impact on the malaria prevalence in human (Fig. S2.8).


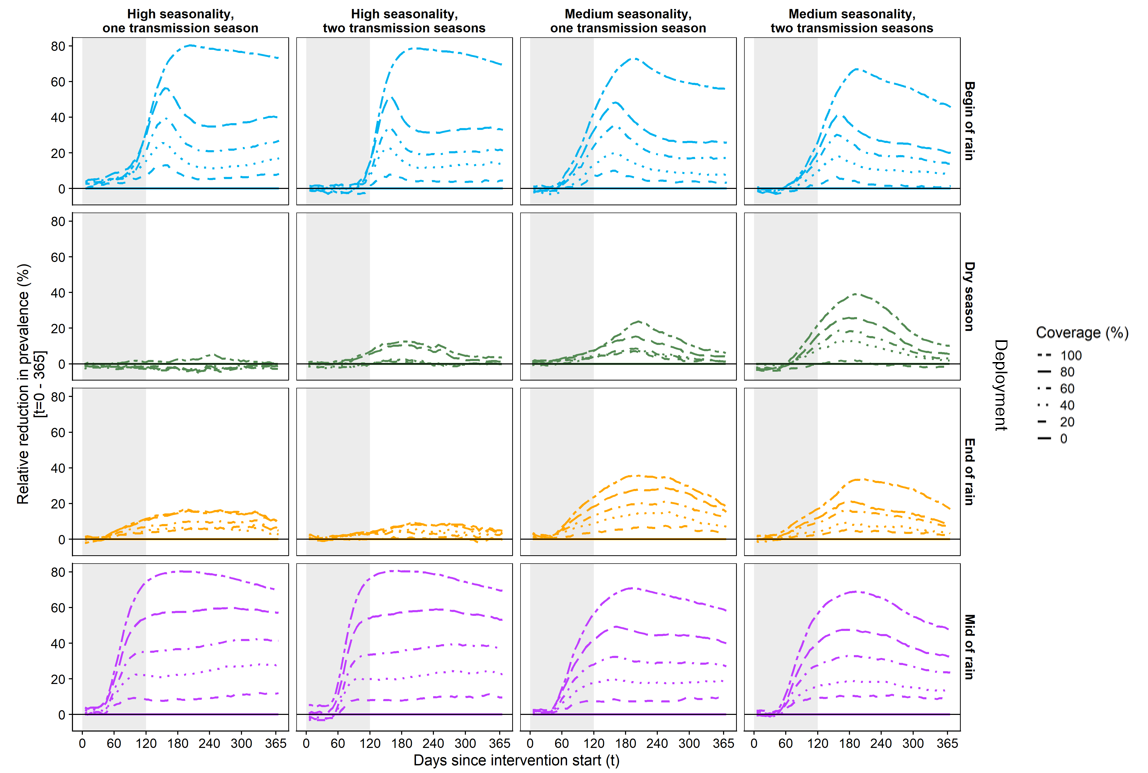


Fig. S2.7: **Relative reduction in prevalence over time for varying starting months of larviciding and varying coverages at moderate transmission intensity (EIR=10 ibpa).**

The grey shaded area indicates the time larviciding was deployed assuming constant effectiveness. The colored lines show the starting months as selected for the defined seasonal deployment of larviciding.


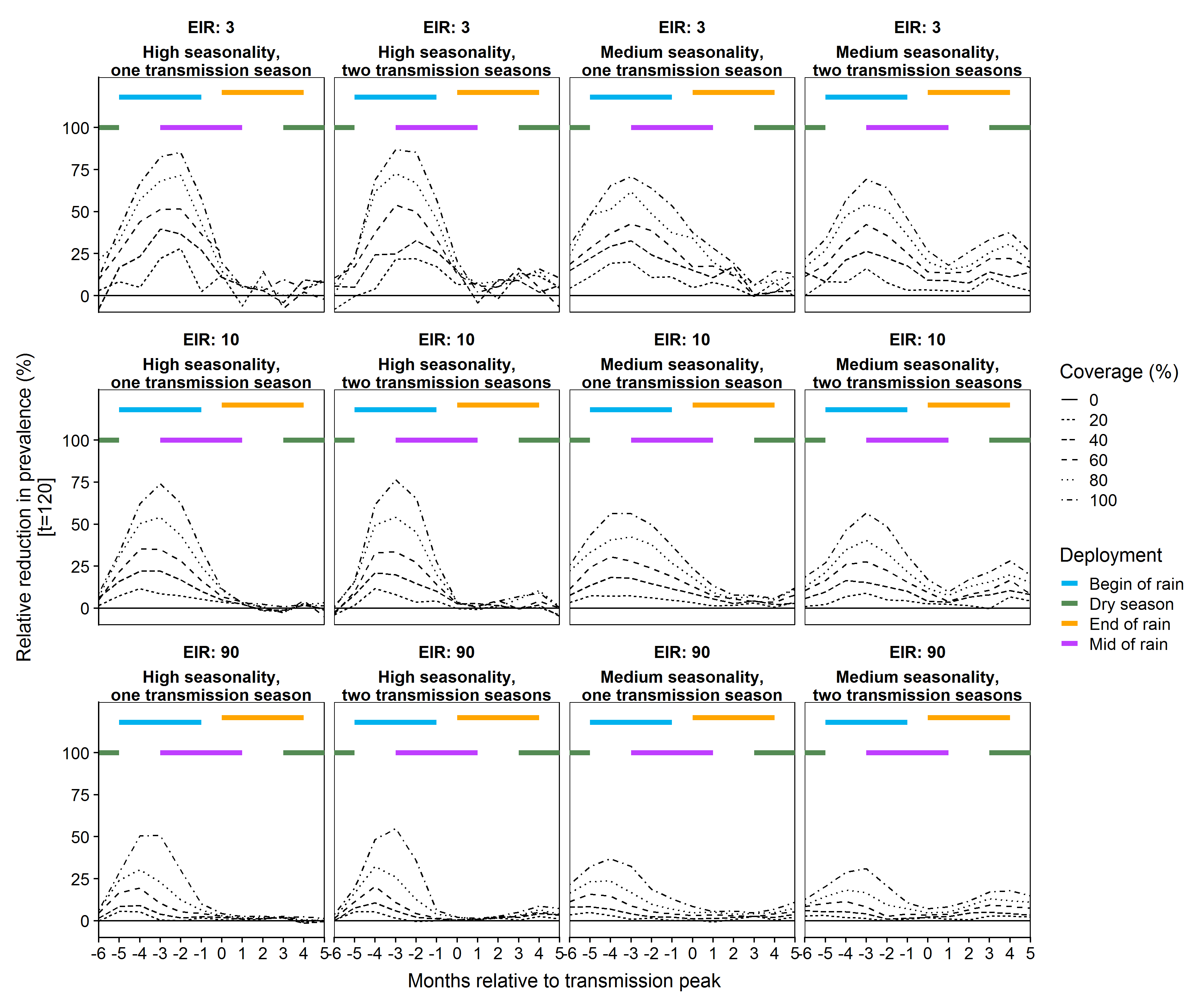


Fig. S2.8: Monthly shift in deployment and relative reduction in prevalence with varying coverage and transmission intensity.

The colored lines indicate the seasonal timing explored (begin of rain = -6 to -4 , dry season = 3 to 5, end of rain=0 to 2, and mid of rain=-3 to -1 months before the peak in transmission).


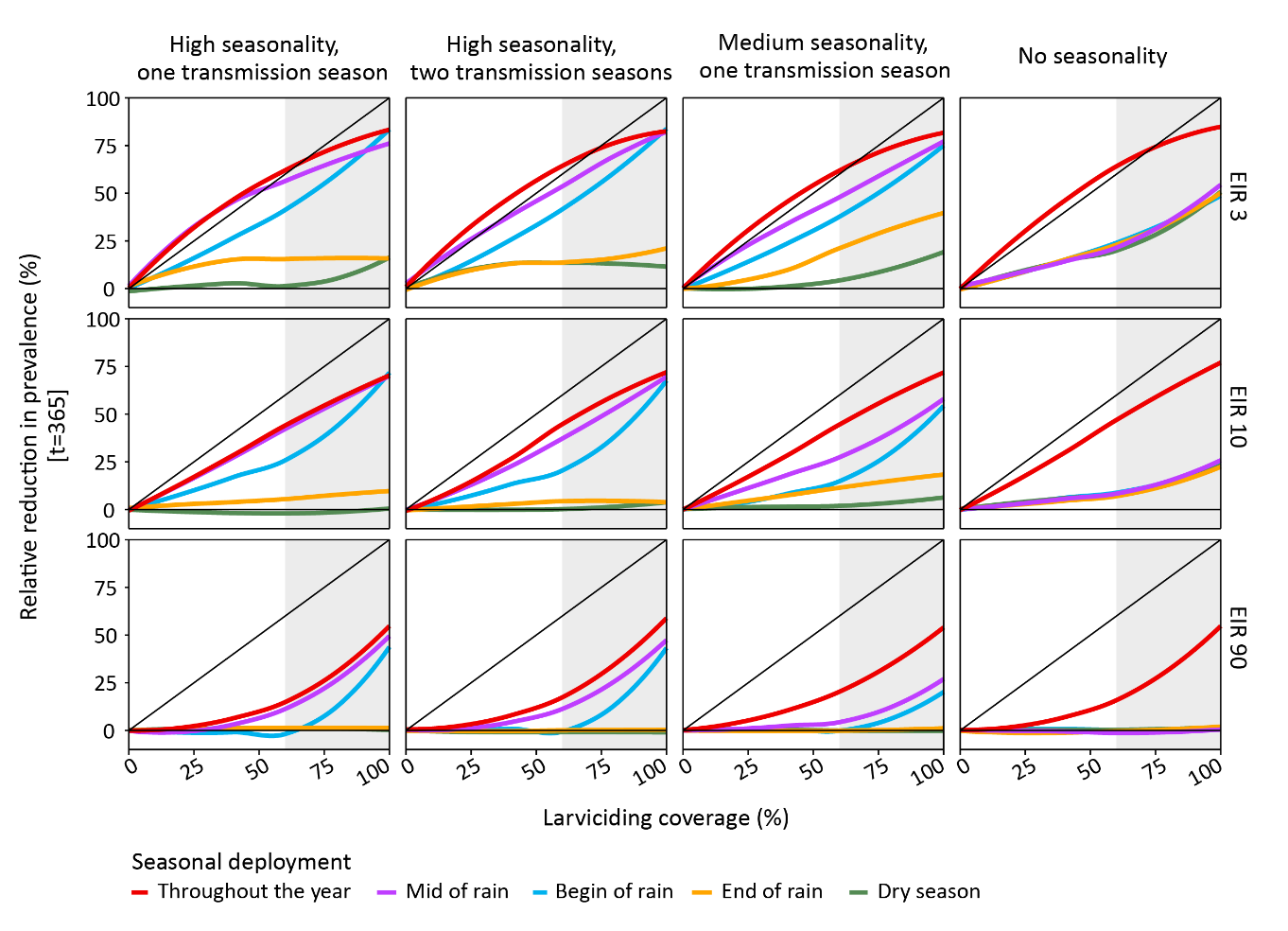


Fig. S2.9: Simulated relative reduction in prevalence, after one year of larviciding deployment, by coverage and timing of deployment for different seasonality and intensity of transmission.

The x-axis shows the effective larviciding coverage. The grey area shows effective larviciding coverage above 60%, which might be difficult to achieve in practice. The coloured shaded areas represent the 95% confidence interval for the relative reduction for different timing of deployment, assuming a fixed deployment period of one year (throughout the year) or 120 days (seasonal deployment).

## Coverage thresholds and seasonal timing

At the end of the intervention period, the maximum relative prevalence reduction of larviciding during the wet season ranged between 69.8% and 47.8% depending on seasonality with higher reductions at high seasonality and one peak. In this setting (high seasonality and one peak) the prevalence reduction ranged from 0.5% for larviciding applied at maximum coverage during the dry season to 73.2% during the beginning of the wet season. The difference depending on timing was lowest in settings with medium seasonality and two peaks (RR_dry season_=10.4%, RR_wet season_=47.8%). The maximum reduction in perennial settings (constant transmission) would be 26.5% for 120 days and 76.8% for 365 days of effective larviciding (Table S2.1).


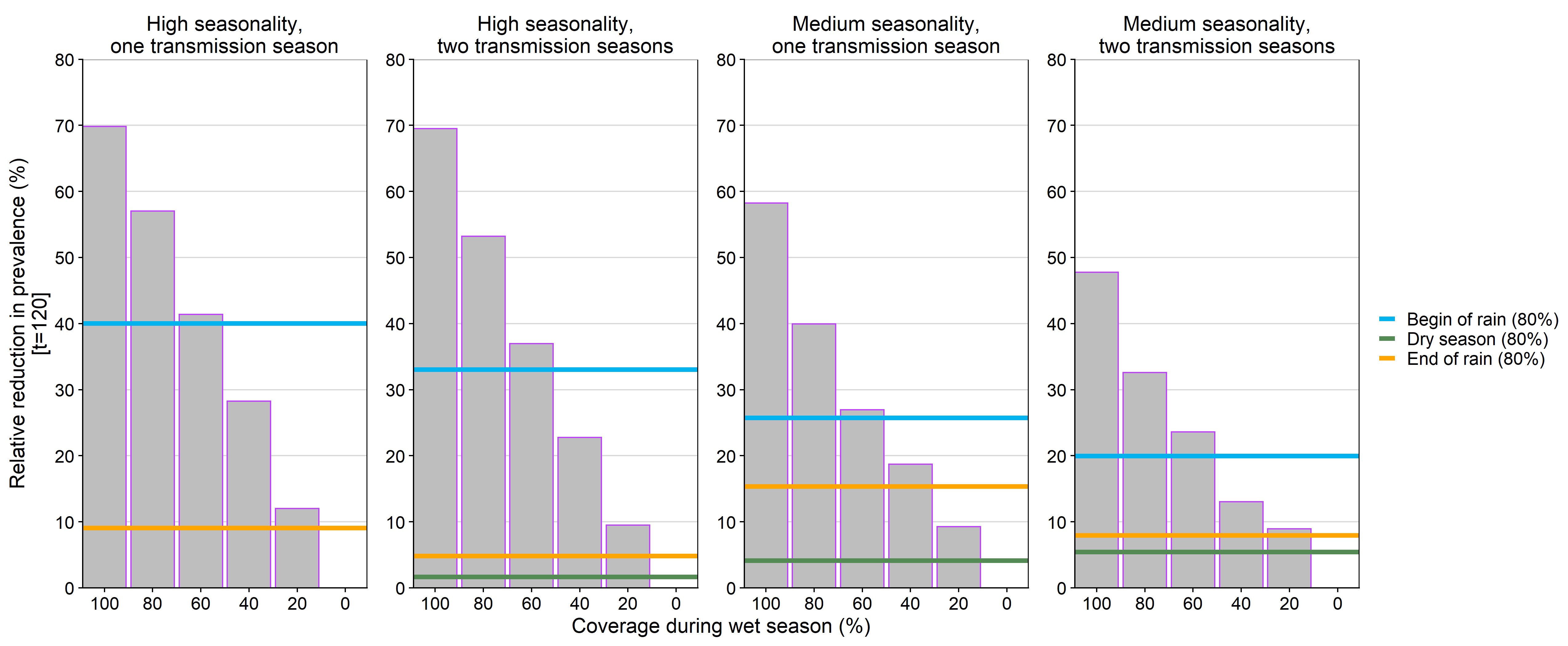


Fig. S2.10: Threshold for larviciding coverage during the wet season compared to an arbitrary effective target coverage of 80% during the other seasons after larviciding with constant effectiveness throughout deployment period of 120 days at moderate transmission (EIR= 10 ibpa).

Table S2.1: Relative mean reduction in prevalence at moderate transmission (EIR=10 ibpa) with varying seasonality and deployment times after 120 days effective larviciding.

|  | |  | **Start of larviciding, effective for four months** | | | | **Throughout the year** |
| --- | --- | --- | --- | --- | --- | --- | --- |
| **Seasonality** | **Peaks** | **Coverage** | **Begin of rain** | **Dry season** | **End of rain** | **Mid of rain** |  |
| None |  | 20 | 2.5 | 4.2 | 3.1 | 2.6 | 13.1 |
| Medium | one | 20 | 3.3 | 1.6 | 4.2 | 9.3 | 13.7 |
| Medium | two | 20 | 1.5 | -1.6 | 2.3 | 8.9 | 16.5 |
| High | one | 20 | 8.3 | -1.5 | 4.9 | 12.0 | 12.0 |
| High | two | 20 | 4.4 | 0.0 | -0.2 | 9.5 | 9.4 |
| None |  | 40 | 6.4 | 5.7 | 4.4 | 5.7 | 30.0 |
| Medium | one | 40 | 7.4 | 1.8 | 7.0 | 18.7 | 27.9 |
| Medium | two | 40 | 7.6 | 1.9 | 3.3 | 13.0 | 33.0 |
| High | one | 40 | 17.1 | -1.1 | 2.6 | 28.2 | 29.0 |
| High | two | 40 | 14.5 | 0.1 | 4.1 | 22.7 | 26.5 |
| None |  | 60 | 9.4 | 8.0 | 7.2 | 8.9 | 46.8 |
| Medium | one | 60 | 17.0 | 1.2 | 11.6 | 27.0 | 44.7 |
| Medium | two | 60 | 14.1 | 2.9 | 6.7 | 23.6 | 48.9 |
| High | one | 60 | 26.9 | -2.8 | 5.5 | 41.4 | 44.2 |
| High | two | 60 | 21.4 | -0.1 | 3.6 | 37.0 | 44.9 |
| None |  | 80 | 12.6 | 11.8 | 12.2 | 13.1 | 63.2 |
| Medium | one | 80 | 25.7 | 4.1 | 15.4 | 39.9 | 59.9 |
| Medium | two | 80 | 20.0 | 5.4 | 7.9 | 32.6 | 62.9 |
| High | one | 80 | 40.0 | -1.1 | 9.1 | 57.0 | 57.6 |
| High | two | 80 | 33.0 | 1.7 | 4.8 | 53.2 | 60.5 |
| None |  | 100 | 24.8 | 23.5 | 22.7 | 26.5 | 76.8 |
| Medium | one | 100 | 55.8 | 6.1 | 18.2 | 58.2 | 71.5 |
| Medium | two | 100 | 45.6 | 10.4 | 17.0 | 47.8 | 76.6 |
| High | one | 100 | 73.2 | 0.5 | 9.2 | 69.8 | 70.4 |
| High | two | 100 | 69.4 | 3.7 | 3.8 | 69.5 | 71.7 |

1. <https://github.com/SwissTPH/openmalaria/wiki/ScenarioTransmission> [↑](#footnote-ref-1)
2. Service MW. Medical entomology for students. 5th ed. Cambridge: Cambridge University Press; 2012. [↑](#footnote-ref-2)
