# Supplementary material for: Evaluation of different deployment strategies for larviciding to control malaria: a simulation study
Source: Malar J. 2021 Jul 27;20:324. doi: 10.1186/s12936-021-03854-4 (PMC8314573; doi:10.1186/s12936-021-03854-4)
Supplement: Supplementary file 3 — Additional file 3: Flowchart from operational to effective larviciding coverage. [file 12936_2021_3854_MOESM3_ESM.pdf]

Additional File 3: Flowchart from operational to effective larviciding coverage

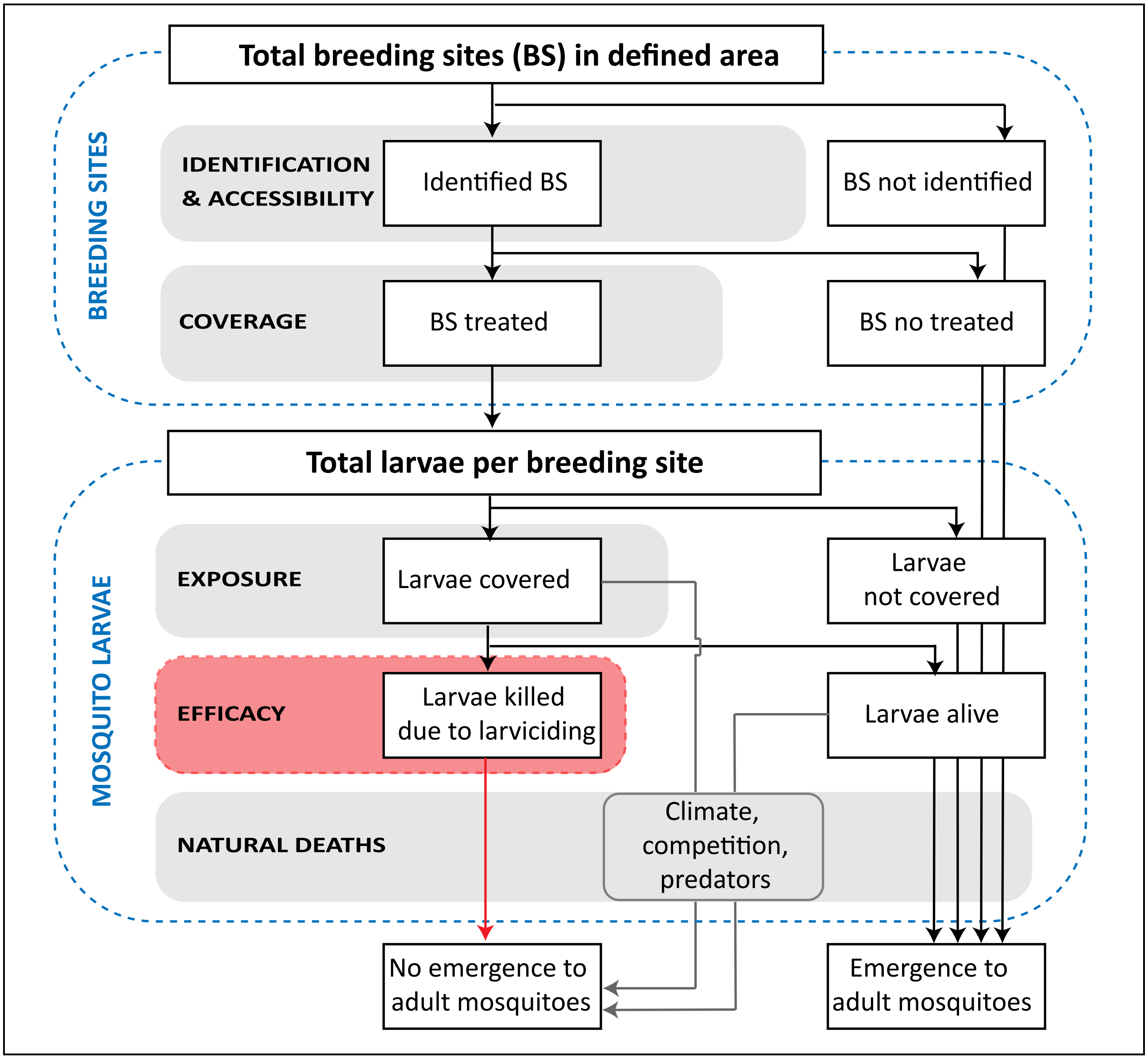

The figure reads from top to bottom, capturing coverage factors between identification of a breeding site and effective emergence reduction. **IDENTIFICATION & ACCESSIBILITY**: Out of all productive breeding sites, only a subset of such is identified and accessible, depending on the performance of larviciding staff as well as environmental conditions. **COVERAGE**: Out of the identified and accessible breeding sites either all or only a subset might be treated, due to factors related to either staff performance, or study design, as some studies only treat breeding sites in which larvae were found to be present. This coverage is referred to <u>operational coverage</u> and the only measurable coverage (number of breeding sites treated out of all (identified) breeding sites. **EXPOSURE**: Within breeding sites, it might be that the larvicide is not applied evenly on the breeding site surface, due to either staff performance or environmental conditions, leading to the possibility that not all larvae within a breeding site get exposed to the larvicide. **EFFICACY**: The efficacy describes the effect rate of the larvicide on exposed larvae, and some larvae might survive despite being exposed, due to environmental factors or decay of the larvicide, this refers also to the <u>effective coverage</u> as used in the modelling. **EMERGENCE REDUCTION**: the proportion of larvae killed due to being exposed to the larvicide; it is a combination of the effective coverage and the larvicide efficacy.
