# Supplementary material for: Evaluation of different deployment strategies for larviciding to control malaria: a simulation study
Source: Malar J. 2021 Jul 27;20:324. doi: 10.1186/s12936-021-03854-4 (PMC8314573; doi:10.1186/s12936-021-03854-4)
Supplement: Supplementary file 4 — Additional file 4: Re-simulated larviciding study in Mbita, western Kenya between 2002 and 2006. [file 12936_2021_3854_MOESM4_ESM.docx]

**Additional File 4: Re-simulated larviciding study**

# Study summary

Between 2002 and 2005 Fillinger and colleagues conducted a larviciding field study in a rural village in Kenya (Mbita). At that time, ITNs and IRS had not been scaled up [1]. Malaria transmission in Mbita was described as perennial, with two rainy seasons: long rains from March to June and shorter rains from October to December. Larviciding was applied throughout the year between June 2002 and September 2004 using *Bacillus sphaericus* (*Bs*) and *Bacillus thuringiensis var. israelensis* (*Bti*). In total, 419 breeding sites were reported, with around half of those containing *Anopheles* larvae, and 65-219 breeding sites treated per larviciding deployment (n=50 deployments) (varying habitat availability per deployment) [1].

Table S4.1: Number of deployments separated by larvicide used. Reproduced from Fillinger et al. [1].

| ***Bs.*** | | | |  | ***Bti.*** | | | |
| --- | --- | --- | --- | --- | --- | --- | --- | --- |
| **No** | **Date** | **Breeding habitats treated** | **Days between treatments** |  | **No** | **Date** | **Breeding habitats treated** | **Days between treatments** |
| 1 | 13.06.2002 | 101 | – |  | 8 | 07.11.2002 | 165 | 22 |
| 2 | 24.06.2002 | 88 | 10 |  | 14 | 30.01.2003 | 94 | 22 |
| 3 | 09.07.2002 | 67 | 16 |  | 16 | 02.04.2003 | 185 | 58 |
| 4 | 23.07.2002 | 78 | 14 |  | 17 | 09.04.2003 | 120 | 7 |
| 5 | 12.08.2002 | 76 | 20 |  | 18 | 16.04.2003 | 219 | 7 |
| 6 | 03.09.2002 | 66 | 22 |  | 19 | 23.04.2003 | 180 | 7 |
| 7 | 16.10.2002 | 108 | 43 |  | 20 | 01.05.2003 | 155 | 8 |
| 9 | 13.11.2002 | 189 | 6 |  | 21 | 07.05.2003 | 178 | 6 |
| 10 | 26.11.2002 | 153 | 13 |  | 22 | 14.05.2003 | 194 | 7 |
| 11 | 18.12.2002 | 144 | 22 |  | 23 | 21.05.2003 | 192 | 7 |
| 12 | 22.12.2002 | 206 | 4 |  | 24 | 28.05.2003 | 126 | 7 |
| 13 | 08.01.2003 | 145 | 17 |  | 28 | 10.12.2003 | 172 | 25 |
| 15 | 05.02.2003 | 73 | 6 |  | 29 | 17.12.2003 | 110 | 7 |
| 25 | 04.06.2003 | 123 | 7 |  | 30 | 24.12.2003 | 120 | 7 |
| 26 | 01.07.2003 | 132 | 27 |  | 31 | 31.12.2003 | 83 | 7 |
| 27 | 15.10.2003 | 72 | 105 |  | 32 | 07.01.2004 | 75 | 7 |
| 34 | 21.01.2004 | 93 | 7 |  | 33 | 14.01.2004 | 145 | 7 |
| 35 | 11.02.2004 | 68 | 21 |  | 37 | 31.03.2004 | 156 | 27 |
| 36 | 04.03.2004 | 82 | 22 |  | 38 | 07.04.2004 | 191 | 7 |
| 47 | 09.06.2004 | 79 | 7 |  | 39 | 14.04.2004 | 198 | 7 |
| 48 | 15.07.2004 | 68 | 36 |  | 40 | 21.04.2004 | 200 | 7 |
| 49 | 03.09.2004 | 65 | 50 |  | 41 | 28.04.2004 | 202 | 7 |
| 50 | 15.09.2004 | 135 | 12 |  | 42 | 05.05.2004 | 166 | 7 |
|  |  |  |  |  | 43 | 12.05.2004 | 139 | 7 |
|  |  |  |  |  | 44 | 20.05.2004 | 102 | 8 |
|  |  |  |  |  | 45 | 26.05.2004 | 85 | 6 |
|  |  |  |  |  | 46 | 02.06.2004 | 76 | 5 |

# Simulations and analysis

Simulations in OpenMalaria were run to compare the predicted effect of larviciding to outcomes of the larviciding study conducted in Mbita [1]. In the simulations, the annual baseline transmission intensity, the seasonality, vector species, time of the larviciding applications and the larvicide used were selected as reported in the study and the coverage was varied. The assumed effectiveness decay was twenty days for *Bs* and ten days for *Bti* using step function for simplicity. The simulations were run with three seeds for a population of 10000 people without importation of infections. A “warm-up” period of 60 years before the implementation of larviciding was run to reflect on-going transmission intensity. The predictions were aggregated per months and relative reductions calculated as described in the main document.

Table S4.2: Specified setting and deployment parameters

|  | **Simulation 1** | **Simulation 2** |
| --- | --- | --- |
| **Simulation setup** |  |  |
| Scenarios | 363 | 393 |
| Population | 10000 | 10000 |
| Seeds | 3 | 3 |
| **Setting** | | |
| Transmission intensity (annual EIR) | 10 | 10 |
| Vector species | *An. gambie s.s* | *An. gambie s.s* |
| Monthly seasonality  (approximated by rainfall) | 238, 100, 283, 500, 471, 240, 131, 126, 164, 145, 316, 319 | 238, 100, 283, 500, 471, 240, 131, 126, 164, 145, 316, 319 |
| Importation | None | None |
| **Larviciding** | | |
| Deployment time | As reported  (see Table A4.1) | Fixed (constant during intervention period) |
| Decay of larvicides | Specific to *Bs* or *Bti* as reported  (see Table 1) | No distinction between larvicide  Fixed to be identical to the deployment time |
| Larviciding coverage | Varied, separately per larvicide  *Bti* = 0-100%  *Bs* = 0-100% | Varied, for both larvicide combined, but varied per season  *Dry* = 0-100%  *Wet* = 0-100%  All year round = 0-100% |

# Result figures


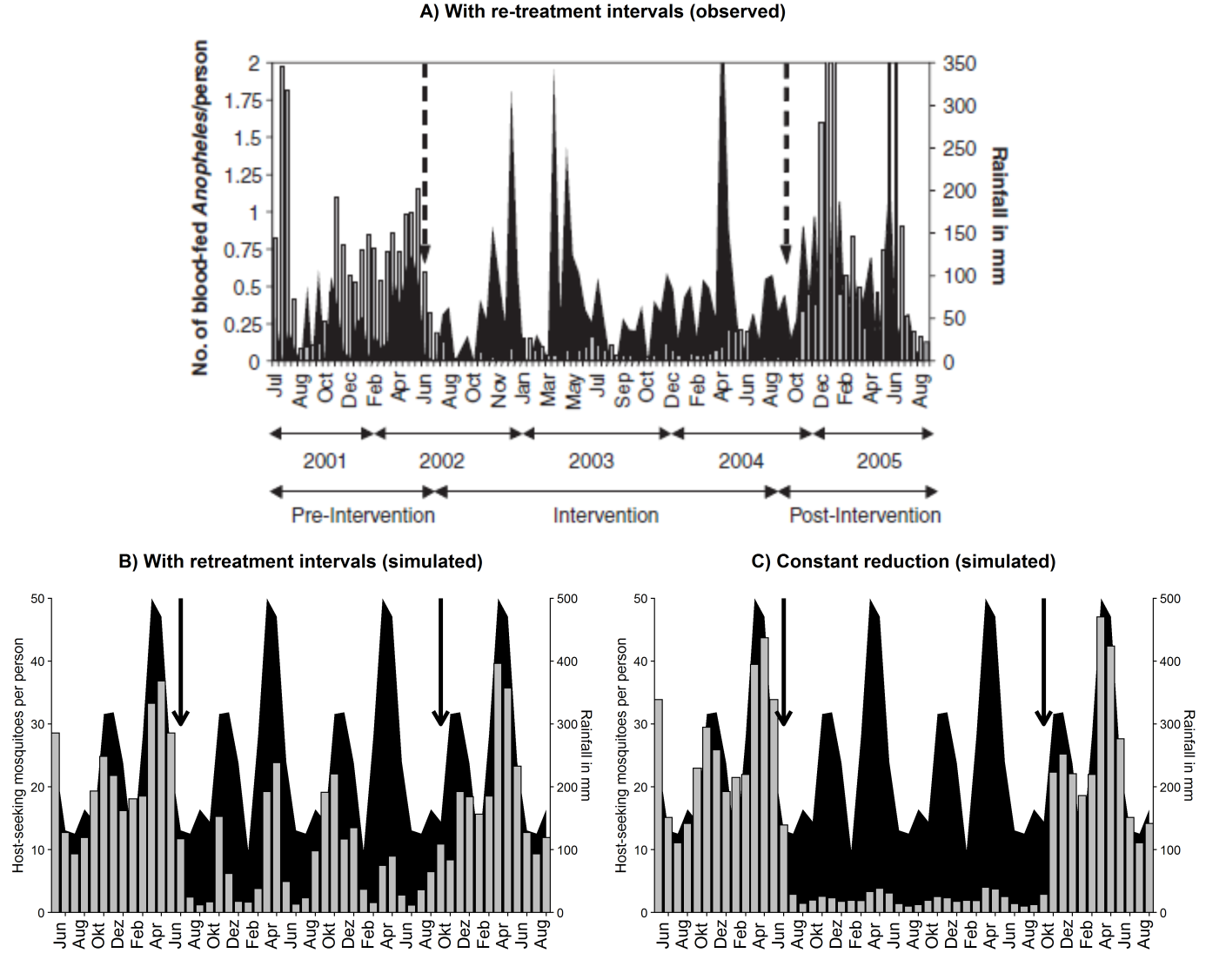


Fig S4.1: Visualised observed and simulated reduction in adult mosquitoes due to larviciding. A) Reprinted results from Fillinger et al. 2006 [1], with larviciding impact on blood fed mosquitoes caught indoors. The black area indicates the seasonality and the bars the larvae density, the colour of the bars type of mosquito. B-C) Simulated impact of larviciding with 90% coverage on mosquito emergence per population with B) exact decay and deployment as reported in the study and C) constant effectiveness throughout the intervention period. The scales need to be interpreted in relative terms, as the caught number of mosquitoes was much lower than the number of simulated mosquitoes per person for an EIR of 10 infectious bites per person.

**References**

1. Fillinger U, Lindsay SW. Suppression of exposure to malaria vectors by an order of magnitude using microbial larvicides in rural Kenya. Trop Med Int Health. 2006;11:1629–42.
